# Transcriptomic characterization of transitional B cells reveals four subsets with perturbed activation profiles in scleroderma

**DOI:** 10.1016/j.isci.2026.117504

**Published:** 2026-09-15

**Authors:** Claire Beesley, Nina Goldman, Gisela Gabernet, Edel Aron, Cankut Çubuk, Myles J. Lewis, Louisa K. James, Gur Yaari, Steven H. Kleinstein, David J. Abraham, Christopher P. Denton, Rizgar A. Mageed, Voon Ong

**Affiliations:** 1 Division of Medicine, Centre for Rheumatology, University College London, London, UK; 2 Department of Pathology, Yale School of Medicine, New Haven, CT 06520, USA; 3 Centre for Experimental Medicine and Rheumatology, William Harvey Research Institute, Barts and the London School of Medicine and Dentistry, Queen Mary University of London, London, UK; 4 Centre for Immunobiology and Infection, Faculty of Medicine and Dentistry, Blizard Institute, Queen Mary University of London, London, UK; 5 Program in Computational Biology and Biomedical Informatics, Yale University, New Haven, CT, USA; 6 Centre for Translational Medicine and Therapeutics, Queen Mary University of London, London, UK; 7 College of Pharmacy, Al-Kitab University, Kirkuk 36015, Iraq

**Keywords:** B cells, autoimmunity, systemic sclerosis

## Abstract

We previously demonstrated that patients with scleroderma or systemic sclerosis (SSc) have elevated autoreactive transitional B cells, including against topoisomerase I (anti-topoisomerase I autoantibody [ATA^+^]). This suggests that defective transitional B cell tolerance could drive autoimmunity in SSc. To investigate this, we used single-cell transcriptomic and B cell receptor (BCR) sequencing on sorted transitional B cells from treatment-naive SSc patients and healthy controls (HCs). Four transitional B cell clusters were identified as T1, T2, CD27^+^, and marginal zone precursors (MZPs). T1 B cells were significantly expanded in SSc with a 2-fold increase compared with HCs. Additionally, pro-survival genes (IL4R, TCL1A, and S100A10) and IFN-responsive genes (IFITM1 and IFITM2) were upregulated in SSc. BCR analysis revealed increased complementarity determining region 3 (CDR3) hydrophobicity and altered κ/λ ratios with proximal Jκ gene usage in the SSc patients. Collectively, our findings support divergent transitional B cell development and activation in SSc with an AKT-driven pathway likely promoting autoreactive transitional B cell survival.

## Highlights

• Human transitional B cells are classified into T1, T2, CD27^+^, and MZP
• SSc patients manifest significant expansion of transitional T1 B cells
• SSc transitional B cells show upregulation of pro-survival and IFN-responsive genes
• The SSc transitional B cell repertoire is distinct from HCs

## Graphical abstract

**Figure:**
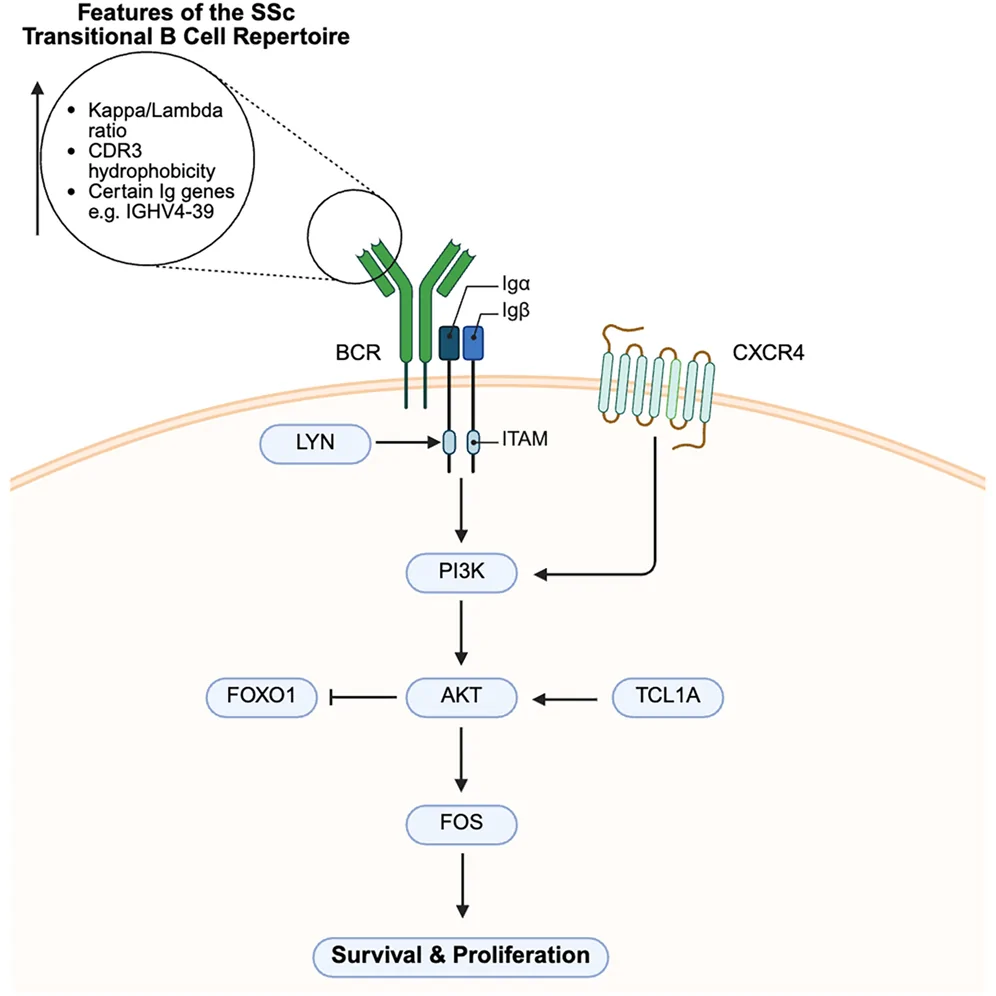

## Teaser

Health sciences; Medicine; Transcriptomics

## Introduction

There is compelling evidence that B cells play a key role in systemic sclerosis (SSc) pathogenesis.^1^ This is supported by clinical studies of rituximab, a B cell depleting monoclonal antibody specific for CD20, and more recently with emerging promising results using CD19-targeting chimeric antigen receptor (CAR) T cell therapy in a small number of cases.^2^^,^^3^^,^^4^ B cells secrete antibodies and cytokines that are essential for the coordination of protective immune responses and can initiate immune dysregulation, namely through the production of T-dependent and T-independent autoantibodies. Importantly, B cells can also instigate autoimmunity by presenting autoantigens to activate autoreactive T cells.^5^ In SSc, antinuclear autoantibodies are central to diagnosis and differentially predict risk of organ-based complications.^6^ Furthermore, growing evidence suggests that some SSc-specific autoantibodies may promote fibrosis.^7^ In addition, recent studies suggest that anti-topoisomerase I autoantibodies (ATAs) can penetrate live cell nuclei and inhibit topoisomerase I activity.^8^ This supports the hypothesis that ATAs contribute to significant cellular dysfunction in SSc. We have previously identified that an immature subset of B cells, termed transitional B cells, are autoreactive in SSc patients and produce ATAs at an early stage of their development.^9^ These cells are absent in healthy individuals and ATA seronegative patients, suggesting that defective B cell tolerance mechanisms could permit the survival of ATA^+^ B cells in SSc.

Transitional B cells are immature lymphocytes that have recently migrated to the periphery from the bone marrow. They are present in the circulation as well as secondary lymphoid organs, including the spleen and gut-associated lymphoid tissue where they undergo maturation to follicular or marginal zone B cells.^10^ It is thought that there are at least three transitional B cell populations termed T1, T2, and T3, representing sequential stages of early B cell development as they transition to mature B cells.^10^ T1 cells represent the earliest transitional B cells that have migrated from the bone marrow and are highly susceptible to apoptosis following engagement of their B cell receptor (BCR).^11^ T2 cells represent an intermediate stage with increased survival and potential for further maturation, while T3 cells are considered anergic B cells with limited contribution to the effective mature B cell pool.^11^^,^^12^

Human transitional B cells can be identified by flow cytometry using a combination of either CD24 and CD38 or CD10 and CD21 cell-surface proteins.^11^ Refined enrichment of the individual transitional subsets can be achieved with the inclusion of CD27, IgM, IgD, and ABCB1.^11^ In this study, we enriched transitional B cell subsets using antibodies against CD19, CD24, CD38, CD27, IgM, and IgD. Using this gating strategy CD19^+^CD24^hi^CD38^hi^ cells are considered as the total transitional B cell pool and the CD27^−^ T1–T3 subsets are contained within this pool. Of relevance to this study, CD19^+^CD24^hi^CD38^hi^ cells are sometimes used as a proxy for regulatory B cells. This may relate to the increased production of IL-10, observed within CD19^+^CD24^hi^CD38^hi^CD27^+^ B cells, and transitional marginal zone precursor (MZP) cells, which are thought to be contained within IgM^hi^ T2 cells.^13^^,^^14^^,^^15^^,^^16^

Previous studies demonstrated that transitional B cells resist apoptosis upon BCR engagement in SSc and could thereby escape effective peripheral tolerance.^9^ These findings were corroborated by a later study from Glauzy et al.^17^ who reported alterations in the transitional B cell compartment in SSc patients and demonstrated that mechanisms of central and peripheral B cell tolerance are likely compromised in SSc.^17^ Furthermore, the authors reported that there was a significantly higher frequency of long complementarity determining region 3 (CDR3) regions in the transitional B cell repertoire of SSc patients compared with healthy controls (HCs).^17^ This is important because the CDR3 region is the most variable region of the BCR and has a major impact on antigen specificity. As such, specific properties of the CDR3 region have been associated with increased propensity for self-binding, including longer CDR3s and more hydrophobic composition.^18^^,^^19^^,^^20^ To better understand the relevance of transitional B cells in SSc, we assess the transcriptome and B cell repertoire of SSc patients and HCs. We propose that transitional B cells are central to the initiation of autoimmunity in SSc and that better understanding of tolerance breakdown will underpin more effective and better tolerated cell targeted therapy in the future.

## Results

### Identification and transcriptional characterization of transitional (T1, T2, and MZP) and CD27^+^ B cell subsets

**Figure 1:**
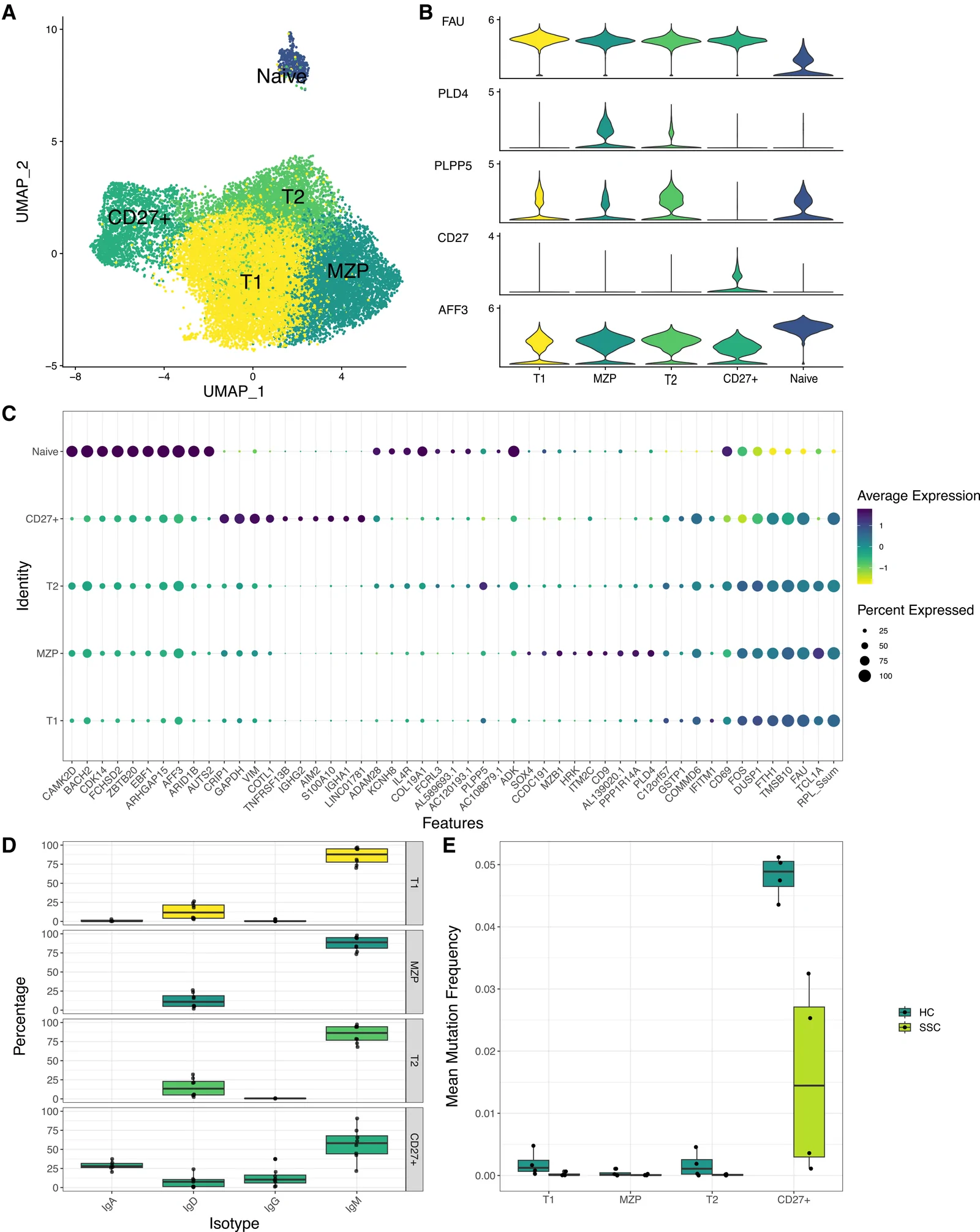
Single-cell characterization of sorted B cells enriched for the CD19^+^CD24^hi^CD38^hi^ B cell pool (A) Uniform manifold approximation and projection (UMAP) plot to show annotated T1, T2, CD27^+^, marginal zone precursors (MZPs), and naive B cells. (B) Violin plot to highlight key annotation genes per cluster. (C) Dotplot showing the top genes per cluster as identified by FindAllMarkers. RPL_Ssum represents the total expression of genes encoding large and small ribosomal proteins. (D) Isotype usage as % of B cells expressing that isotype in the different clusters. (E) Mean mutation frequency per donor across the clusters in HCs and SSc patients.

Using single-cell immune profiling to characterize CD19^+^CD24^hi^CD38^hi^ transitional B cells in four diffuse treatment-naive ATA^+^ SSc patients (median age 50; median disease duration 9 months) and four HCs (demographics in Table 1), we identified five distinct B cell clusters as depicted in Figure 1A and annotated them according to canonical marker genes (Figures 1B and 1C). We conducted these initial characterization analyses using only the HC cells to avoid any disease-associated transitional B cell signature, as described further. High expression of *TCL1A*, an AKT coactivator, has previously been associated with transitional and naive B cells.^21^ In agreement with this, we identified robust *TCL1A* expression in 71%–88% of cells in the HC T1/T2/MZP clusters and reduced expression in the HC naive cluster (39% of cells). We also identified a prominent ribosomal gene signature in our data with ribosomal genes, such as *RPL30* and *RPL41*, accounting for 33%–39% of the total gene expression in the T1, T2, MZP, and CD27^+^ clusters from the HCs. This is consistent with a prior study that demonstrated enhanced ribosome biogenesis in transitional and follicular B cells.^22^ Additionally, we identified shared expression of 886 genes between the T1, T2, and MZP and CD27^+^ clusters in the HC samples (Figure S3A). Subsequent analysis revealed that pathways related to translation and metabolism were among the top 10 enriched pathways in HC transitional clusters using the Kyoto Encyclopedia of Genes and Genomes (KEGG) and Reactome databases (Figure S3B).

**Table 1:** Clinical and demographic features of the 5′ scRNA-seq and bulk RNA-seq patient cohort

	SSc scRNA-seq	SSc bulk only	HC
Total (*n*)	4	2	4
Female (%)	50 (2)	100 (2)	75 (3)
Age (years)	50 (42–70)	48 (30–66)	67 (36-78)
Disease duration (months)	9 (3–12)	6.5 (6–7)	–
mRSS	14.5 (8–33)	8.5 (8–9)	–
Organ involvement (%)			
Interstitial lung disease	75 (3)	100 (2)	–
Muscle	25 (1)	50 (1)	–
Cardiac	25 (1)	0	–

All SSc patients were diffuse, treatment naive, and ATA^+^. Three donors (1 SSc patient and 2 HCs) were included in both the scRNA-seq and bulk RNA-seq. Two additional SSc patients were included in the bulk RNA-seq only. Results presented as *n* (%) or median and interquartile range. Abbreviations: modified Rodnan skin score (MRSS) and interstitial lung disease (ILD).

As anticipated, we observed a high degree of similarity between the T1 and T2 cluster in HCs with expression of genes involved in regulating cellular processes, such as protein synthesis (*FAU*), cytoskeletal proteins (*TMSB10*), iron metabolism (*FTH1*), and stress-signaling pathways (*DUSP1*). Compared with the T1 cluster, T2 cells had elevated expression of the lipid phosphatase *PLPP5* and *IGHD*, associated with maturing B cells.^23^ Conversely, the MZP cluster expresses marginal zone associated genes, such as *CD9*, *PLD4*, and *MZB1*.^13^^,^^24^^,^^25^ The CD27^+^ cluster expresses *CD27* in 28% of cells, as well as the metalloproteinase *ADAM28*, the cytoplasmic DNA sensor *AIM2*, and the actin-binding protein *COTL1*. Additionally, *TNFRSF13C (BAFF-R)* expression gradually increased from T1 to MZP to T2 and CD27^+^ cells, while the BAFF receptor *TNFRSF13B (TACI)* was predominantly found in the CD27^+^ cluster. BAFF-R is an important pro-survival receptor in B cells and is expressed by transitional B cells to rescue them from premature cell death^26^ In contrast, the naive cluster has increased expression of B cell transcription factors, such as *EBF1*, and is likely a contaminant from the cell sorting.

Furthermore, Ig isotype distribution as identified through analysis of the single-cell VDJ data confirms the identities of these cell types with the majority of transitional B cells (defined as T1, T2, MZP, or CD27^+^) expressing *IGHD* or *IGHM* on the VDJ assay (Figure 1D). Somatic hypermutation (SHM) frequencies in these populations were low (below 0.01 mutations per base pair) although there are some cells with higher SHM frequencies in the CD27^+^ cluster (Figure 1E). B cell clones were also analyzed and limited clonal expansion was observed as expected for immature B cells. However, overall clonal diversity was increased in SSc across the Hill diversity indices (q = 0, 1, and 2) with the most notable elevation in the T1 cluster. This suggests increased clonal richness and reduced clonal dominance in the transitional SSc repertoire.

Interestingly, the previously proposed anergic T3 cluster was not detected in the single-cell RNA sequencing (scRNA-seq) dataset, although we observed that 30% of HC cells in the MZP cluster expressed the pro-apoptotic gene *HRK*. Additionally, some anergy-related genes, such as *CD72*, *PTEN*, and *PTPN6* or *SHP-1*, were enriched in the MZP cluster compared with the other transitional clusters. For example, *CD72* was expressed in 51% of HC MZP cells, compared with an average of 29% in other HC transitional clusters. These genes were also expressed, but in a lower proportion of cells in other clusters, suggesting that B cell anergy may be inducible throughout transitional B cell maturation as previously suggested.^22^

We also used bulk RNA-seq to analyze RNA extracted from sorted CD24^hi^CD38^hi^ T1 and T2 transitional B cells as we hypothesized that there may be a B cell tolerance checkpoint at this stage, as has been previously reported.^27^ However, after completing differential gene expression analyses between T1 and T2 cells, very few genes passed the threshold for significance when comparing T1 with T2 (Figure S4), again indicating that these cell types are similar. Of these genes, *RNU1-1*, *ADAM23*, and *XAB2* were significantly upregulated in the T1 cluster and the genes *IGHV4-34* and *CLCC1* were significantly upregulated in the T2 cluster. This is in comparison with the 362 differentially expressed genes in the scRNA-seq data, suggesting that the sorting strategy used for the bulk RNA-seq experiment may not have optimally resolved the T1 and T2 subsets.

We next sought to verify the cluster annotation genes for the T1, T2, and MZP clusters by using a publicly available dataset from Stewart et al*.*^21^ Importantly, this study used a different gating strategy to sort transitional B cells from one healthy donor (CD19^+^IgD^+^CD27^−^CD10^+^, rather than CD19^+^CD24^hi^CD38^hi^ cells used in our study) and had excluded CD27^+^ cells. While the authors concluded that they could not sub-cluster the transitional B cells, we were able to identify distinct T1, T2, and MZP clusters using our annotation genes (Figures S5A and S5B). Consistent with our data, the T1 cluster comprised the largest proportion of cells and we identified a prominent ribosomal gene signature accounting for an average of 34% expressed genes across the clusters. Additionally, expression of the pro-apoptotic *HRK* gene, alongside anergy-related *PTPN6* and *CD72*, were highest in the MZP cluster corroborating our data.

### T1 B cells are expanded in SSc patients while CD27^+^ cells are reduced

**Figure 2:**
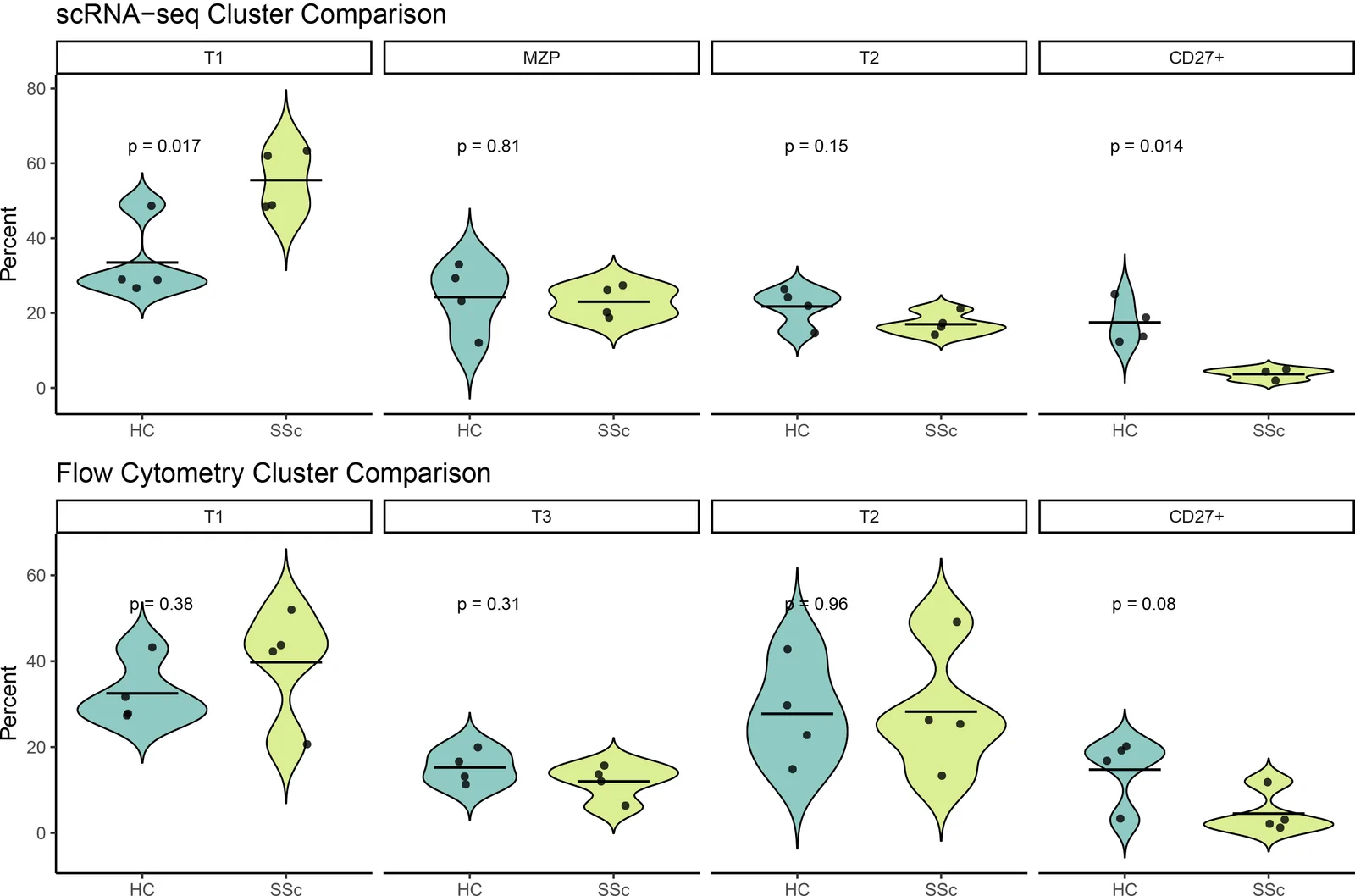
Distributions of transitional B cell clusters as determined by paired flow cytometry and scRNA-seq Mean proportions of cells per cluster and sample as identified via scRNA-seq (top) and flow cytometry (bottom). Statistical analysis was carried out using a Student’s *t* test.

Differences in transitional B cell numbers and proportions in SSc patients have been previously reported.^9^^,^^17^ We, therefore, sought to assess the frequency of transitional B cell subsets through our paired flow cytometry and scRNA-seq data (Figure 2). This demonstrated an increase of T1 B cells in both datasets, although the difference was only significant in the scRNA-seq data where the proportion of T1 cells was nearly 2-fold higher in the SSc patients (a mean percentage of 55.5% of total transitional cells in the SSc patients compared with 33.5% in the HCs). Additionally, in both the scRNA-seq and flow cytometry data there was a significant reduction in the proportion of CD27^+^ cells in the SSc patients compared with the HCs.

### The MZP cluster shares similarities with T3 cells and previously described IgM^hi^ T2 cells

It should be noted that the T3 cells identified using flow cytometry were not found in the single-cell analysis (Figure 2). However, the T3 subset may represent the MZP cluster in the scRNA-seq data. This is because the MZP cluster has the highest level of genes associated with B cell anergy and apoptosis, phenotypes that were previously ascribed to T3 cells, alongside robust expression of marginal zone associated genes *PLD4*, *CD9*, and *MZB1*, as described earlier. Furthermore, T3 B cells are inconsistently described in the literature with some groups defining them as anergic B cells and others as late-transitional naive B cell precursors. Hence, as we could identify expression of marginal zone associated genes, we designated the cluster MZP cells, rather than T3, although it is possible that these two subsets could overlap. This is supported by the proportional analysis in Figure 2 as the percentage of MZP cells in the scRNA-seq data and T3 cells in the flow cytometry data is similar. Hence, there is no MZP subset in the flow cytometry data and no T3 cluster in the scRNA-seq clusters. Interestingly, MZP cells have previously been identified in a subset of human IgM^hi^ T2 cells that differentiate from T1 precursors^13^ and have higher expression of *ITGB7*, as well as lower expression of IL-4 receptor (*IL4R*) than IgM^lo^ T2 cells. In our data, the percentage of IgM^+^ cells is 88.5% in the HC T2 cells and 92.5% in the MZP cells. The MZP cells also have significantly lower expression of *IL4R* and an increased proportion of cells expressing the gut-homing integrin *ITGB7* than the T2 cluster. Hence, we hypothesize that the previously identified IgM^hi^ T2 subset could correspond to the MZP cluster in our data.

### Trajectory analysis predicts that a key cell-fate decision occurs at the T1 stage

**Figure 3:**
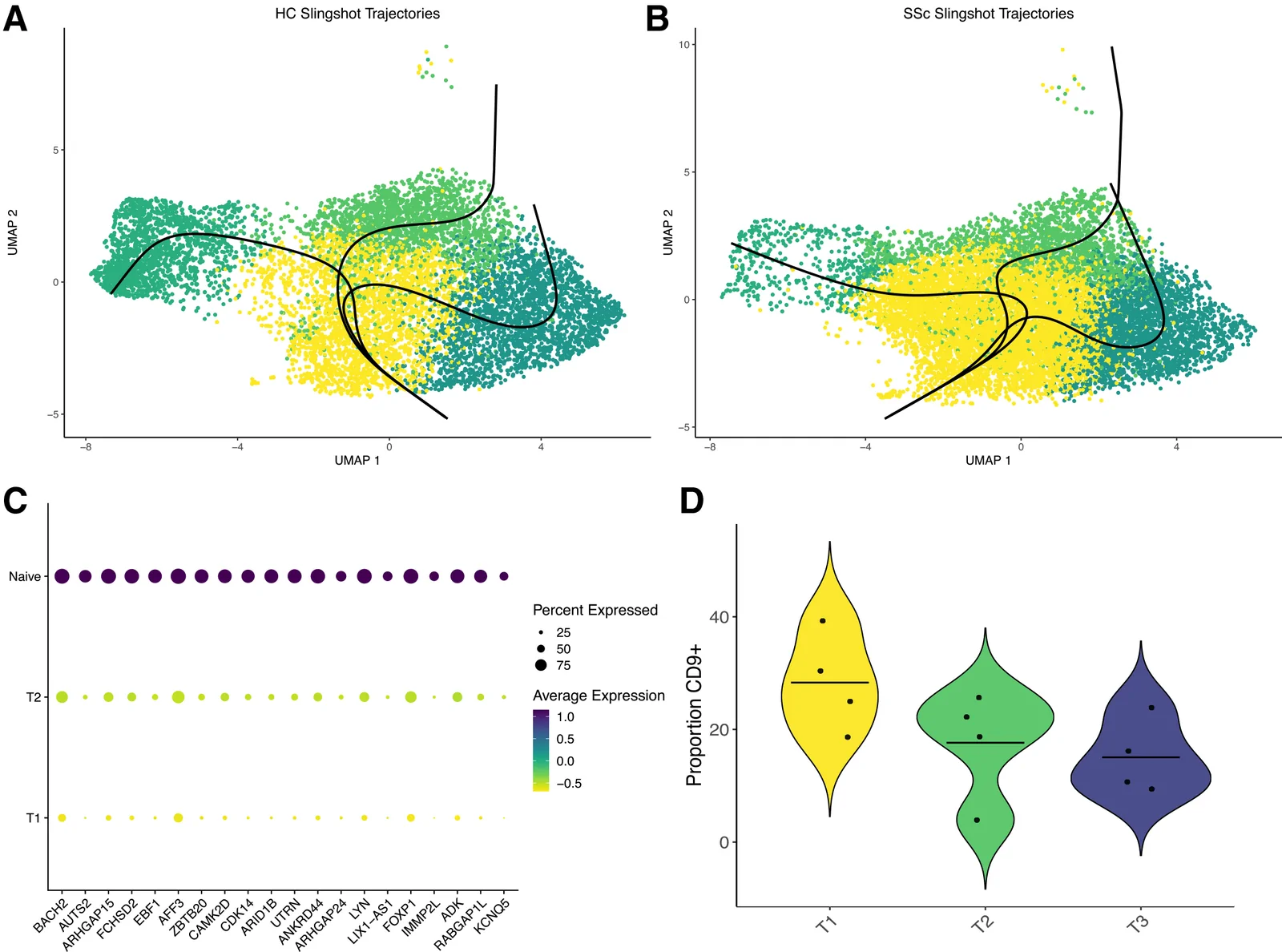
Trajectory inference predicts three distinct transitional B cell fates (A) Trajectory analysis of the HC samples (*n* = 4). (B) Trajectory analysis of the SSc samples (*n* = 4). (C) The top 20 annotation genes in the naive B cell cluster shown in the T1, T2, and naive clusters. (D) Flow cytometry analysis of CD9 protein expression, a gene associated with the MZP cluster in the scRNA-seq data, in T1, T2, and T3 B cells in HC samples.

To infer the relationships between the clusters, we performed trajectory analysis on the HC samples with Slingshot^28^ (Figure 3A). This analysis suggests that a key cell-fate decision is likely to occur within the T1 cluster, a possibility that is supported by the trajectory analysis of the SSc patients (Figure 3B). Importantly, these analyses are consistent with the previously described data by Tull et al.^13^ in which the authors identified a bifurcation of human B cell maturation at the T1 stage leading to follicular and marginal zone B cells. To support the well-characterized follicular T1-T2 trajectory in our data, we identified that the expression of naive B cell annotation genes gradually increases from T1 to T2 and into the naive cluster (Figure 3C). Meanwhile, to support the divergent T1-MZP trajectory we analyzed flow cytometry expression of CD9 in transitional B cells from four additional HC samples. This is because CD9 was expressed by 40% of cells in the MZP cluster in HCs and has been associated with murine MZ B cells (Figure 3D).^25^ Although not statistically significant, the flow cytometry data revealed that the frequency of CD9^+^ cells was highest in the T1 cluster compared with the T2 and T3 clusters, potentially supporting the notion that T1 cells could directly give rise to MZP cells. We additionally identified a third trajectory from the T1 to CD27^+^ cluster. This was an unexpected finding as CD24^hi^CD28^hi^CD27^+^ cells have typically been ascribed as Bregs or IgM^+^ memory B cells and their relation to T1 cells has not been determined. Therefore, to assess the potential function of the CD27^+^ cluster we used a publicly available dataset of sorted human IgM memory B cells (CD19^+^IgD^+^CD27^+^) from Stewart et al.^21^ to compile a list of IgM memory B cell annotation genes (Figure S2). Interestingly, the CD27^+^ cluster had an elevated IgM memory B cell score compared with the other clusters, which was most evident in the HCs (Figure S6, top). Similarly, using a publicly available Breg dataset from Baig et al., the CD27^+^ cluster revealed an elevated proportion of cells with a high Breg score compared with the other clusters, which was again most evident in the HCs (Figure S6, bottom).^29^ These findings suggest the CD27^+^ cluster may represent a pool of IgM memory B cells and Bregs. This is consistent with previous studies demonstrating the regulatory capacity of CD24^hi^CD38^hi^CD27^+^ cells and suggesting that there may be overlap between CD24^hi^CD38^hi^ transitional Bregs and IgM memory Bregs. Additionally, the finding that the CD27^+^ cluster is enriched with a population of cells with a high Breg score, suggests that the T1 to CD27^+^ trajectory could represent a novel pathway for Breg induction. In support of this possibility, T1 B cells have the highest percentage of CD27 expressing cells and additionally express the highest level of CD27 compared with the T2 and MZP clusters. Although, not statistically significant, CD27 expression was also increased in the T1 cells in the bulk RNA-seq dataset compared with the T2 cells, providing further support for a potential induction of CD27 at the T1 stage. Furthermore, T1 cells also have the second highest Breg score after the CD27^+^ cluster in the HCs (Figure S6, bottom). These observations remain to be functionally validated.

### A transitionally derived CD27^+^ subset expands in SSc

**Figure 4:**
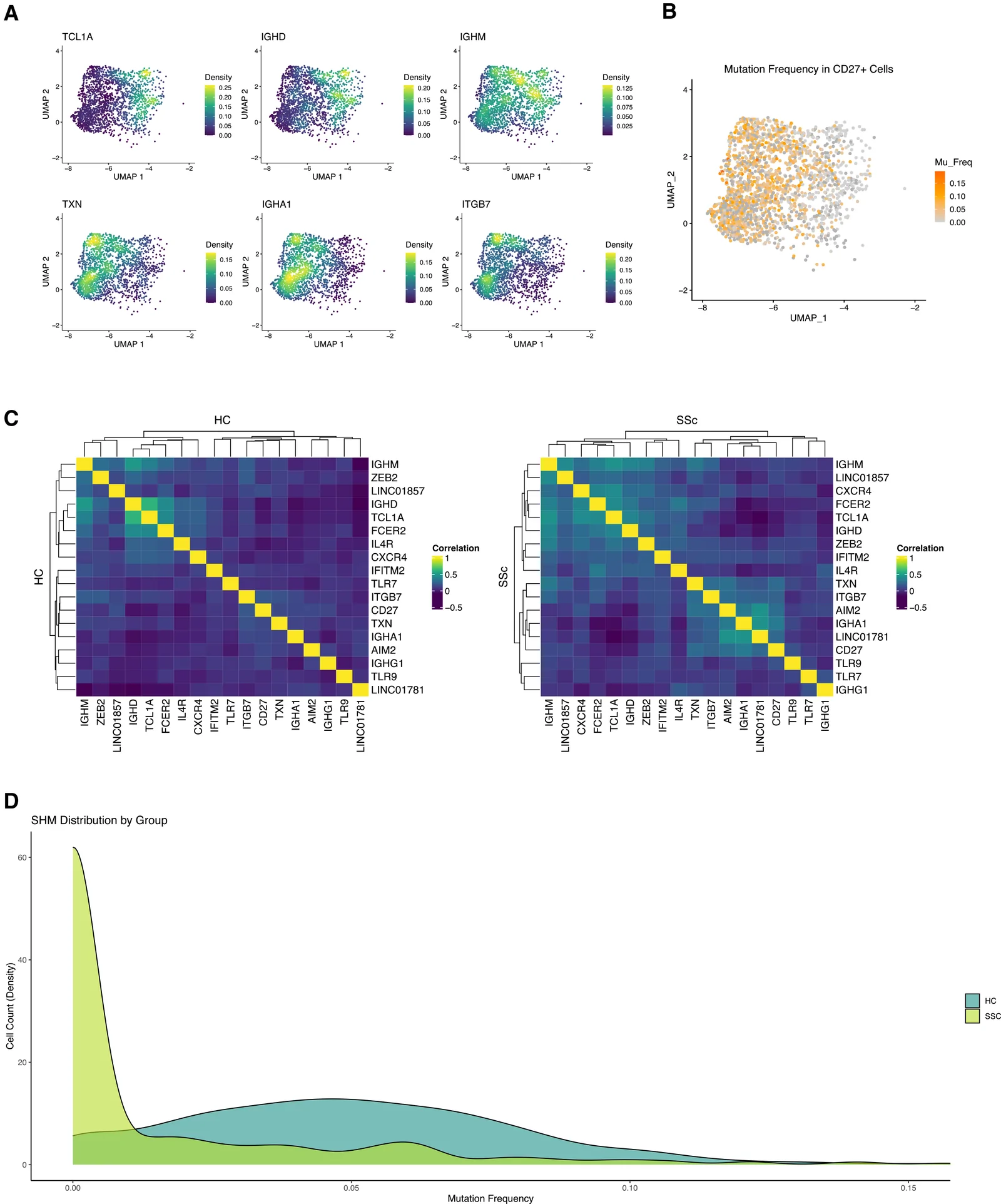
In-depth characterization of the CD27^+^ cluster (A) Nebulosa plots showing distinct expression patterns of TCL1A/IGHD/IGHM and TXN/IGHA1/ITGB7 in the CD27^+^ cluster. (B) Mutation frequency (Mu_Freq) within the CD27^+^ cluster. (C) Heatmap correlation plot of differentially expressed genes in the CD27^+^ cluster in the HCs (left) and SSc patients (right). (D) Somatic hypermutation (SHM) distribution and comparison in the CD27^+^ cluster.

As the CD27^+^ cluster contains cells with higher average SHM frequency, we hypothesized that this cluster may be heterogeneous, containing both mutated and unmutated B cells. In agreement with this, cells with high SHM lacked expression of the transitional marker *TCL1A* and expressed genes associated with gut-homing and Breg phenotypes, including *ITGB7* and *TXN* (Figures 4A and 4B).^30^^,^^31^ Correlation analysis confirmed this heterogeneity (Figure 4C) showing correlated expression of naive-associated genes, such as *TCL1A*, *IGHM*, *IGHD*, *FCER2*, and *IL4R*, while *CD27* correlated with *TXN*, *IGHA1*, and *ITGB7*. We also identified a significant expansion of unmutated CD27^+^ cells in SSc (Figure 4D) potentially suggesting increased recruitment of T1-derived cells into this compartment.

### B cell activation and survival genes are upregulated in transitional B cells from SSc patients

**Figure 5:**
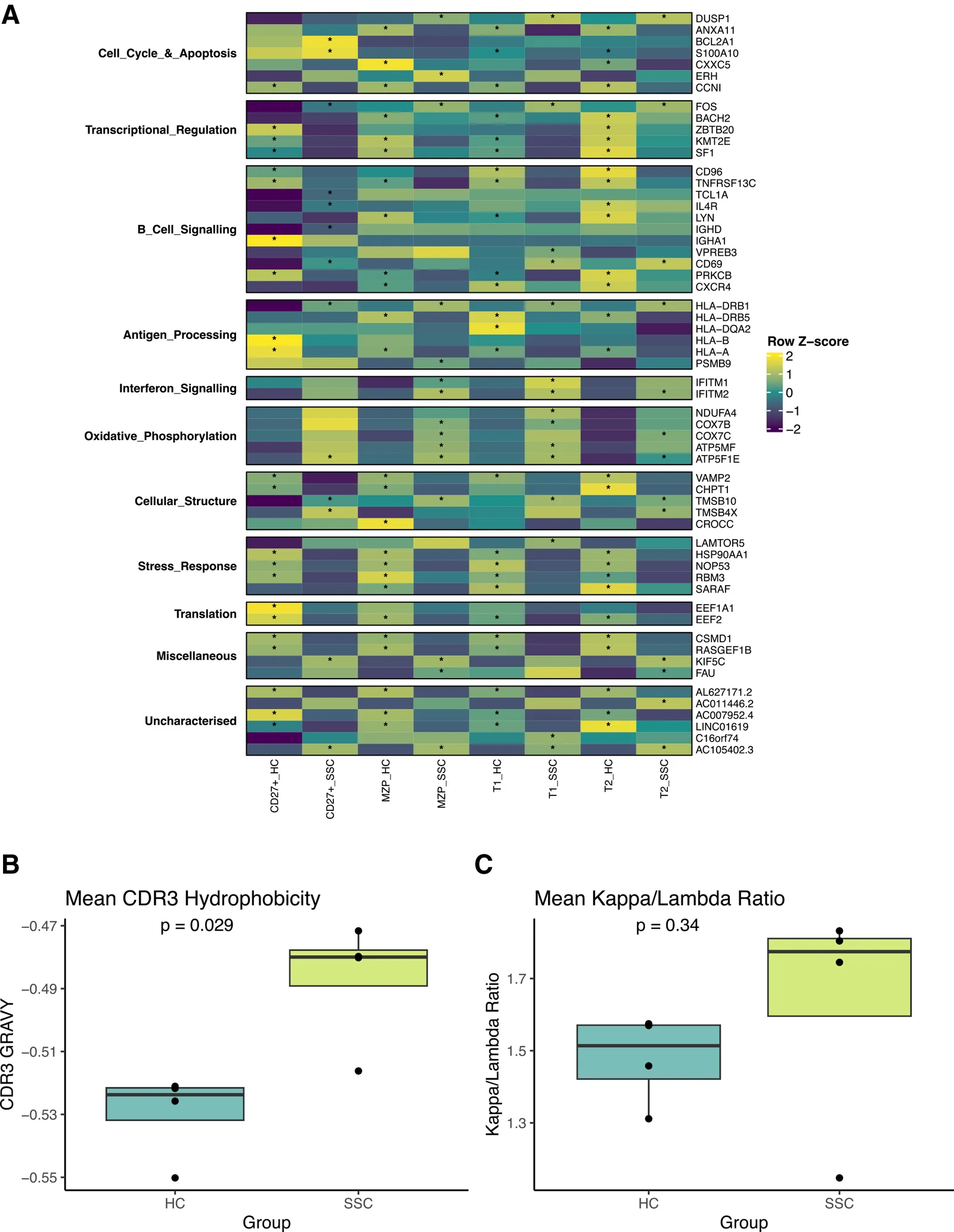
Distinct activation profiles and alterations in the transitional B cell repertoire of SSc patients (A) Heatmap showing log-normalized pseudobulk expression values for the top 13 differentially expressed genes per group and cluster identified in transitional B cell clusters from SSc patients compared with HCs. Differentially expressed genes were identified using Wilcoxon rank-sum testing. For visualization, pseudobulk expression was calculated by averaging gene expression within each cluster and group. Rows represent differentially expressed genes grouped by gene function. The asterisks denotes where the gene was differentially expressed and are placed in the column representing the direction of upregulation. (B) Mean CDR3 gravy or hydrophobicity score per donor in total transitional B cells from SSc patients compared with HCs. Groups were compared with a Wilcoxon rank-sum test. (C) The mean light-chain kappa/lambda ratio per donor for transitional B cells in SSc patients compared with HCs. Groups were compared with a Wilcoxon rank-sum test.

We have previously demonstrated that transitional B cells in SSc contain a high level of autoreactive cells that produce elevated levels of pro-inflammatory cytokines, such as IL-6.^9^ To assess the transcriptomic basis for these functional anomalies we performed differential gene expression analyses per cluster between HCs and SSc patients in our scRNA-seq data (Figure 5A; Tables S3 and S4). We identified several relevant genes that are dysregulated in SSc and may be related to enhanced B cell activation and inflammatory cytokine production (Figure 5A). For example, the AP-1 transcription factor complex *FOS* is significantly elevated in transitional B cells from SSc patients and high levels of *FOS* have previously been associated with plasma cell differentiation in cancer,^32^ indicating that elevated *FOS* may be relevant for autoantibody production. Additionally, B cell activation genes, such as the interferon responsive genes *IFITM1* and *IFITM2*, were upregulated in SSc, consistent with an elevated disease-specific interferon signature. In contrast, expression of genes involved in regulating B cell activation (*LYN* and *BACH2)* was reduced. These genes are important for restraining antigen-receptor signaling and have been implicated in other autoimmune diseases, such as systemic lupus erythematosus (SLE).^33^^,^^34^^,^^35^^,^^36^^,^^37^^,^^38^ Some of these genes, such as *FOS*, are differentially expressed across all transitional clusters, whereas other genes show cluster-specific differential expression.

Additionally, the forkhead transcription factor, *FOXO1* was downregulated in SSc transitional B cells (Figure S3) along with the long non-coding RNA (lncRNA) *LINC01619* (Figure 5A). This lncRNA regulates *FOXO1* expression by sequestering the microRNA *miR-27a* that negatively regulates *FOXO1*. This is important as *FOXO1* mediates central B cell tolerance by ensuring that autoreactive B cells undergo receptor editing or clonal deletion, opposing the survival-promoting PI3K pathway.^39^

Furthermore, the immune-regulatory gene *CD96* was downregulated in SSc transitional B cells. This gene can regulate T cell and natural killer (NK) cell activity,^40^ but its function has not been described in B cells before. In the HC data, *CD96* was specifically expressed in the T1 and T2 clusters, indicating it may be involved in regulating peripheral B cell tolerance at the T1 to T2 stage.

Another notable finding was the upregulation of *TNFRSF13C* (BAFF-R) in HC transitional B cells. In contrast, *IL4R* that encodes the IL-4 receptor was upregulated in the SSc CD27^+^ cluster. IL-4 is a crucial factor during transitional B cell maturation as it inhibits upregulation of proapoptotic Bim expression.^41^

There was also reduced expression of the transcription factor *KLF2* in the SSc B cells (Figure S3). This gene is important for splenic homing and maintains distribution between the marginal zone and follicular B cell subsets. In mice, KLF2-deficient follicular B cells exhibit elevated innate-like features and share expression of genes normally present in marginal zone B cells.^42^

### Evidence for defective tolerance in SSc from the B cell repertoire

Some of the differentially expressed genes we have identified suggest that peripheral tolerance mechanisms may be impaired in SSc. We next assessed the B cell repertoire to identify whether there were any repertoire features that may be associated with autoimmunity. We observed significantly elevated use of hydrophobic amino acids in the CDR3 region of SSc transitional B cells compared with HCs (Figure 5B). This was most evident in the T2 cluster (Figure S7A), where we also identified a trend toward increased use of *IGHV4-39* (Figure S7B), a gene that has previously been associated with multiple sclerosis and SLE.^43^^,^^44^ Subsequently, we identified a trend toward reduced use of aromatic, bulky, and polar amino acids across the total transitional B cell clusters in SSc patients, together with a slight elevation in mean CDR3 length supporting the previously reported observations by Glauzy et al. (Figure S8).^17^ Isotype usage was not significantly altered between HCs and SSc patients, although we identified a consistent trend toward elevated IgD usage across all clusters in the SSc patients. As IgD expression is acquired during transitional B cell development, this may reflect enhanced maturation and activation in disease.

Alongside impaired peripheral tolerance, the increased proportion of T1 B cells suggests that SSc transitional B cells may be escaping effective central tolerance in the bone marrow. We, therefore, assessed the kappa/lambda ratio as a proxy for receptor editing (Figure 5C). This can be inferred because the effective progression of continued receptor editing utilizes V and J genes that are located further toward the 3′ end of the kappa and lambda loci.^45^^,^^46^^,^^47^ Therefore, the extent of receptor editing can be analyzed by assessing the usage of individual V and J genes and their relative proximity to the 3′ end of the loci. Although not significant, we identified a notable increase in the kappa/lambda ratio in three of the four SSc patients that was most evident in the T1, T2, and MZP clusters. Moreover, patients with an elevated kappa/lambda ratio also displayed increased usage of the upstream Jκ gene IGKJ1. This may suggest that there is reduced utilization of downstream Jκ genes in the patients with the high kappa/lambda ratio compared with HCs (Figure S9). Taken together, these data could suggest that receptor editing is not occurring as extensively in the SSc patients as in the HCs, although experimental work is needed to verify this hypothesis.

## Discussion

Increasing evidence suggests that B cells are central to SSc pathogenesis. Moreover, the presence of autoreactive ATA^+^ B cells in the transitional subset of SSc patients indicates these early B cells are key instigators of autoantibody production.^9^^,^^17^ However, mechanisms underlying autoreactive transitional B cell survival and differentiation remain unclear. Here, we utilized an unbiased approach to characterize transitional B cells from a well-defined cohort of early stage, treatment-naive ATA^+^ SSc patients and HCs. We sorted CD19^+^CD24^hi^CD38^hi^ cells, a population containing transitional B cells, as well as cells with potential regulatory capacity and CD27^+^ cells. The relationship and degree of overlap between these subsets have not been characterized before. To address this, we used scRNA-seq that has allowed us to identify and characterize four distinct transitional clusters (T1, T2, MZP, and CD27^+^). We robustly annotated these cell types and confirmed their annotations with an independent publicly available scRNA-seq dataset. In the SSc patients, the proportion of T1 B cells was increased 2-fold compared to the HCs. This is important as T1 cells are the earliest bone marrow migrant B cells, perhaps, suggesting a defect with early B cell regulation in the bone marrow in SSc. We hypothesize that this could include increased exodus from the bone marrow, B cells exiting the bone marrow prematurely or impaired peripheral tolerance and altered B cell trafficking, for example within the spleen. However, the elevated kappa/lambda ratio and reduction in J1 gene usage in the SSc patients may support the hypothesis that B cells in SSc are exiting the bone marrow prematurely. Furthermore, several genes were differentially expressed in the SSc patients, including increased expression of AKT activator *TCL1A*, as well as reduced expression of the AKT-inhibited gene *FOXO1*. This may suggest that enhanced AKT signaling promotes autoreactive B cell survival in the SSc patients.

We carefully annotated the clusters using previously described transcriptional data. T1 annotation genes were identified across the clusters, which is logical given their recent exit from the bone marrow, hence, it is likely that T1 B cells would upregulate the expression of housekeeping genes, such as those involved in protein synthesis and cytoskeletal organization. Additionally, T2 annotation genes, such as *PLPP5* and *SELL*, have been previously documented, as have genes associated with MZP cells, including *MZB1* and *PLD4*.^13^^,^^23^ This means that we have confidently annotated the T1, T2, and MZP clusters. We did not identify a T3 anergic cluster as previously reported by flow cytometry and functional studies, although we identified expression of the pro-apoptotic gene *HRK* in 29.9% of HC cells in the MZP cluster. This may suggest that an anergic T3 population could exist within the MZP cluster and future studies should determine whether the T3 and MZP cells share overlapping functionality. We also determined that the MZP cluster could represent the IgM^hi^ T2 cluster with marginal zone features, which was described previously by Tull et al*.*^13^

In relation to the CD27^+^ cluster, we demonstrated that a proportion of this cluster expresses gut-homing genes, such as *ITGB7*, and genes that are associated with Bregs, such as *TXN*.^30^ These cells also have higher SHM frequencies indicating that they may have undergone affinity maturation in gut-associated lymphoid tissue and re-entered the circulation as CD19^+^CD24^hi^CD38^hi^CD27^+^ Bregs. Conversely, the CD27^+^ population with low SHM frequency and *TCL1A* expression may represent a true CD27^+^ transitional B cell subset, which is expanded in SSc. Several differentially expressed genes were specific to the CD27^+^ cluster, including *IL4R*, *IGHA1*, *IGHD TCL1A*, and *LINC01781*. In the HCs, expression of *IGHA1* and *LINC01781* was significantly increased, whereas the SSc patients had significantly increased expression of *IL4R*, *IGHD*, and the transitional-associated gene *TCL1A*. Although TCL1A protein is important for early B cell maturation, a recent study demonstrated that TCL1A is also important for tertiary lymphoid structure formation and B cell trafficking,^48^ indicating that TCL1A overexpression may promote autoreactive B cell survival, proliferation, and autoantibody production in SSc. Targeting TCL1A may, therefore, represent a novel treatment strategy in autoimmune disease.^49^

Although the AKT coactivator *TCL1A* was only identified as a differentially expressed gene in the CD27^+^ cluster, we additionally identified significantly reduced expression of *FOXO1* across the T1, T2, and MZP transitional B cell clusters (Figure S3). This is important because FOXO transcription factors can limit cell survival by regulating a range of target genes that promote pro-apoptotic signaling.^50^ FOXO factors are inactivated by AKT-mediated phosphorylation, allowing cell survival, growth, and proliferation. Therefore, the reduction in *FOXO1* across the transitional clusters may suggest enhanced AKT signaling that is likely to contribute in promoting autoantibody production in SSc patients.

Furthermore, we identified increased expression of several oxidative phosphorylation (OXPHOS) genes across the transitional B cell clusters in SSc, such as NDUFA4, COX7B, ATP5MF, and ATP5F1 E, which were all significantly elevated in T1 B cells compared with HCs. This is interesting given that our prior study identified that sorted T1, T2, T3, and CD27^+^ transitional B cell subsets produce ATAs in SSc patients,^9^ suggesting that the elevation of OXPHOS genes could be related to the heightened energy demands of autoantibody producing cells. Furthermore, our prior studies have demonstrated enhanced production of IL-6 by T1, T2, T3, and CD27^+^ transitional subsets.^9^ This could suggest functional skewing across the transitional clusters, which is interesting given that several genes are differentially expressed in all SSc clusters, for example ATP5F1 E.

In this scRNA-seq study, CITE-seq markers were not included, so annotation of cell types was performed using transcriptomic profiles alone. This is an unbiased approach that has been useful in characterizing the CD19^+^CD24^hi^CD38^hi^ transitional B cell pool and, potentially, identifying new markers that could be used to gate transitional B cell subsets by flow cytometry in future studies. Currently, T1–T3 subsets are often gated on subtle differences in IgM and IgD expression, however, we have identified that the proportion of IgM^+^ and IgD^+^ cells was similar between the T1, T2, and MZP clusters, which may explain why individual sorting of the T1 and T2 subsets and subsequent analysis via bulk RNA-seq was less informative than the scRNA-seq analysis.

Overall, this study advances understanding of transitional B cells and their potential contribution to SSc pathogenesis. Moreover, we identified differentially expressed genes, including *FOXO1* and *TCL1A*, which could implicate the AKT/PI3K signaling pathway in promoting autoreactive transitional B cell survival. These are theoretical considerations and more in-depth studies are required to test this hypothesis. Furthermore, we reported new insights into B cell repertoire features that may be associated with SSc and warrant further investigation. These alterations could persist throughout the B cell repertoire and should be investigated in both circulating and tissue-resident B cells across the diverse autoantibody subsets of SSc patients.

### Limitations of the study

While our study included a small number of patients, these were very early cases who had not yet received standard immunosuppressive therapy. Furthermore, all patients were ATA^+^, an autoantibody group known to have strong genetic association, particularly with MHC class II alleles,^51^ thus, providing a more stable immunogenic background. Nevertheless, further confirmation in larger cohorts is required to confirm whether these findings are generalizable across other SSc stages, subsets, and anti-nuclear antibody groups. Although we share data of both male and female SSc patients here, we have been unable to assess the impact of sex in this study due to the small sample size. Future work should also assess the B cell repertoire and transcriptome at disease-involved tissue sites. This would help to determine whether the SSc-associated immunological and transcriptomic features we have observed within circulating transitional B cells are also noted within affected tissues. Additionally, we have identified several differentially expressed genes that are likely to contribute to autoreactive B cell survival and autoantibody production in early B cells. However, these observations should be verified in future mechanistic and functional studies.

## Resource availability

### Lead contact

Further information and requests for resources should be directed to and will be fulfilled by the lead contact, Voon Ong (v.ong@ucl.ac.uk).

### Materials availability

This study did not generate new unique reagents.

### Data and code availability

• Raw and filtered single-cell sequencing data have been deposited with GEO under accession number GSE318206.
• The code is available on GitHub (francesbee9/Transcriptomic-Characterisation-of-TrB-in-SSc).
• Any additional information required to reanalyze the data reported in this paper is available from the lead contact upon request.

## Acknowledgments

This research was supported by 10.13039/501100012041Arthritis UK (grant no. 22534). The authors are additionally grateful to Janani Sivakumaran-Nguyen who assisted with the cell sorting and faculty staff at UCL Genomics who carried out sequencing. BioRender images were generated under publication license DS29DWWAL5.

## Author contributions

C.B. and N.G. collected samples and completed experiments. C.B., G.G., C.C., and E.A. wrote scripts and analyzed data. G.G., M.J.L., L.K.J., G.Y., S.H.K., D.J.A., R.A.M., and V.O. supervized data analysis. R.A.M. and V.O. designed experiments. All authors contributed to data discussion and editing of the manuscript.

## Declaration of interests

S.H.K. receives consulting fees from Peraton.

## STAR★Methods

### Key resources table

**Table:** 

REAGENT or RESOURCE	SOURCE	IDENTIFIER
**Antibodies**		

Anti-CD19 BV785 (Clone HIB19)	BioLegend	Cat no. 302240; RRID:AB_2563442
Anti-CD27 BV711 (Clone O323)	BioLegend	Cat no. 302834; RRID:AB_2563809
Anti-CD38 PerCP-Cy5.5 (Clone HIT2)	BioLegend	Cat no. 303522; RRID:AB_893314
Anti-CD24 PE (Clone eBioSN3)	Invitrogen	Cat no. 501129573
Anti-IgD FITC (Clone IA6-2)	BioLegend	Cat no. 348206; RRID:AB_10612567
Anti-IgM BV605 (Clone MHM-88)	BioLegend	Cat no. 314524; RRID:AB_2562374
Anti-CD9 PE/Cy7 (Clone HI9-a)	BioLegend	Cat no. 312115; RRID:AB_2728255

**Depositeddata**		

5′ scRNA-seq of CD19^+^CD24^hi^CD38^hi^ sorted B cells from HCs and SSc patients	This paper	GSE318206
Human scRNA-seq of CD19^+^IgD^+^CD27^-^CD10^+^ sorted B cells	Stewart et al.^21^	EBI E-MTAB-9544
Homo sapiens reference genome (GRCh38)	Genome Reference Consortium	https://www.ncbi.nlm.nih.gov/grc/human
IMGT reference directory (ImMunoGeneTics information system)	IMGT	https://www.imgt.org/

**Criticalcommercialassays**		

RNeasy Micro Kit	Qiagen	74004

**Software andalgorithms**		

FlowJo v10	FlowJo	https://www.flowjo.com/flowjo/download
GraphPad Prism	GraphPad	https://www.graphpad.com
R Studio v4.3.1	R	https://cran.r-project.org/bin/macosx/
Python v.3.11.5	Python Software Foundation	https://www.python.org
Change-O	Gupta et al.^52^	https://immcantation.readthedocs.io/en/stable/index.html
SHazaM	Gupta et al.^52^	https://immcantation.readthedocs.io/en/stable/index.html
SCOPer	Gupta et al.^53^	https://immcantation.readthedocs.io/en/stable/index.html

### Method details

#### Isolation of peripheral blood mononuclear cells (PBMCs)

PBMCs were isolated from 40 mL blood and frozen in liquid nitrogen until further use. The patient cohort with their clinical characteristics are shown in Table 1. Patients were defined as early-stage and treatment-naive if they were within a year of SSc onset and had not taken any immunosuppressive medications (including disease-modifying anti-rheumatic drugs and steroids). All patients were recruited at the Royal Free Hospital, Center for Rheumatology. Patients fulfilled the 2013 American College of Rheumatology (ACR)/European League Against Rheumatism (EULAR) classification criteria for SSc.^54^ interstitial lung disease was confirmed by high resolution computed tomography (HRCT) imaging. Cardiac involvement was defined as haemodynamically significant cardiac arrhythmias, conduction defects requiring pacemaker or implantable cardioverter-defibrillator, pericardial effusion or congestive heart failure. Skeletal muscle involvement was defined by proximal muscle weakness on physical examination with elevated serum creatine kinases >2 times normal, myopathic changes on electromyogram or abnormal muscle biopsy. The study was approved by the London-Fulham NHS Research Ethics Committee (IRAS ID 279682) and all patients provided written informed consent. Healthy controls were age- and sex-matched and had no history of autoimmune diseases.

#### Fluorescence-activated cell sorting of transitional B cell subsets

Frozen PBMCs were thawed for 2 min in a 37°C water bath before transfer to prewarmed RMPI medium containing 2% FBS. Cells were passed through a 40 μm cell strainer and blocked with Human TruStain FcX (Biolegend). Antibodies used in the flow cytometry panel are shown in Table S1. Staining was performed in MACs buffer (PBS with 2% FBS and 0.5 mM EDTA) for 30 min at 4°C. Cells were sorted on a FACSAria cell sorter at 4°C using a 130 μm nozzle to optimise viability. Total CD19^+^CD24^hi^CD38^hi^ transitional B cells were collected in 50% FBS plus PBS and immediately transferred to UCL Genomics for single-cell processing. CD19^+^CD24^hi^CD38^hi^CD27^-^IgM^hi^IgD^low^ T1 and CD19^+^CD24^hi^CD38^hi^CD27^-^IgM^med^IgD^med^ T2 were sorted into RNAlater for same-day RNA extraction and subsequent bulk RNA-seq processing. The gating strategy used to sort the transitional B cell subsets is shown in Figure S1. Post-sort purity could not be determined due to the rarity of the target populations.

#### Single-cell analysis of CD19^+^CD24^hi^CD38^hi^ cells

##### Library preparation and sequencing

Prior to loading, cells were counted on a LUNA-FL dual fluorescence cell counter to ensure high viability (>90%). For each sample, approximately 10,000 cells per lane were run on a Chromium 10× controller using 5′ chemistry (10× Genomics) with an expected recovery rate of >5,000 cells per lane. Libraries were generated according to the manufacturer’s instructions and run on an Illumina NovaSeq S1 at 50,000 reads per cell for the gene expression libraries and 5000 reads per cell for the V(D)J libraries.

##### Data preprocessing and quality control

Data were processed using the CellRanger pipeline (10× Genomics), aligned to the GRCh38 genome. Scrublet was used to assign a doublet score to the individual cells in Python (v. 3.11.5).

Cells with fewer than 200 genes per cell and greater than 10% mitochondrial reads were removed from the analysis.

V(D)J sequences were processed using the Change-O^52^ pipeline in the Immcantation Docker Image (immcantation/suite:4.5.0).^53^ Raw sequencing reads were aligned to the IMGT germline reference using IgBLAST. Cells which were only expressing light chain genes or cells expressing multiple heavy chain genes were removed from the analysis. B cell clones were defined using SCOPer^53^ v 1.3.0, according to sequences comprising the same IGHV and IGHJ gene segments, same junction length, 90% identity of the junction region nucleotide sequence, and further partitioned by unique light chains.

##### Normalisation, sample integration and clustering

All samples were merged for downstream integration using Seurat’s (v. 4.4) pipeline^55^ described below and performed on a per sample basis. Gene counts were log normalised and the top 2000 variable genes were determined using FindVariableFeatures. For integration, the merged object was split by sample and the functions SelectIntegrationFeatures and FindIntegrationAnchors were used to integrate data. The integrated object was then scaled and immunoglobulin (Ig) genes, including the misannotated Ig genes AC233755.1 and AC233755.2, were removed from the VariableFeatures list. Following this pre-processing, the first 20 principal components and a resolution of 0.2 were used for clustering with the FindClusters function. These clusters were then paired with the V(D)J data using the unique single-cell barcodes. Clusters with at least 70% of cells expressing a BCR were included in the downstream analysis. Uniform Manifold Approximation and Projection (UMAP) was used for visualisation. After completing initial quality control, we identified seven distinct B cell clusters. Upon further analysis, the 421 cells in cluster five were determined to have a very similar transcriptome to the 8443 cells in cluster zero and were, therefore, reannotated as such (Figure S2). We additionally identified a plasmablast cluster with 26 cells which was removed from the analysis due to its small size.

##### Differential gene expression analysis

Seurat’s FindMarkers function (Wilcoxon rank-sum test) was used to identify differentially expressed genes between HCs and SSc patients on a per cluster basis. *p* values were adjusted for multiple testing using Bonferroni correction. Genes passing the significance threshold (adjusted *p*-value <0.05) were considered.

##### Analysis of the B cell repertoire

B cell repertoire analysis included the assessment of immunoglobulin variable (IGHV) region gene usage, somatic hypermutation (SHM) frequencies and CDR3 properties which were assessed using the Immcantation packages SHazaM and Alazakam, respectively.^52^^,^^56^^,^^57^ Custom scripts were developed and used to analyze the Ig light chain kappa (κ)/lambda (λ) ratio in the donors.

#### Bulk RNA-sequencing of T1 and T2 B cells

##### RNA extraction

RNA was immediately extracted from sorted transitional B cells using the Qiagen RNeasy Micro Kit (Qiagen) according to the manufacturer’s protocol. RNA integrity was assessed using an Agilent 4200 TapeStation (Agilent Technologies) and samples with an RNA integrity number (RIN) ≥ 7 were used for library preparation.

##### Library preparation and sequencing

Libraries were prepared using the Watchmaker Polaris Kit according to the manufacturer’s protocol. Sequencing was performed on a NovaSeq 6000 (Illumina) with S1 100 cycle configuration to generate paired end reads with a read depth of 16 million reads per sample. Quality control of sequencing reads was performed using the nf-core/rnaseq bioinformatic pipeline (v3.14.0). Reads were aligned to the human reference genome (GRCh38) using STAR aligner.

##### Differential gene expression analysis

Bulk RNA-sequencing data was processed using DESeq2^58^ to identify differentially expressed genes between the transitional T1 and T2 populations. Genes with an adjusted *p*-value < 0.05 were considered significant.

### Quantification and statistical analysis

Flow cytometry data was analyzed using FlowJo (v. 10) and GraphPad Prism. For all analyses, a *p*-value < 0.05 was considered statistically significant. Exact tests are denoted in figure legends.

## Supplemental information

Document S1. Figures S1–S9

Table S1. Antibodies used during flow cytometry and fluorescence-activated cell sorting of transitional B cell subsets

Table S2. IgM memory B cell annotation genes identified through analysis from Stewart et al.^28^ This paper sorted for IgM memory B cells from one healthy donor (CD19^+^IgD^+^CD27^+^). We analyzed this dataset, identifying two memory B cell clusters with a clustering resolution of 0.2. We then used FindAllMarkers to identify a list of genes that were positively expressed in these clusters to compile an IgM memory B cell score of the 62 annotation genes identified.

Table S3. A list of the differentially expressed genes between HCs and SSc patients on a per cluster basis identified through FindMarkers

Table S4. A table of the top 13 differentially expressed genes in the HC and SSc patients on a per cluster basis
