# Supplementary material for: Transcriptomic characterization of transitional B cells reveals four subsets with perturbed activation profiles in scleroderma
Source: iScience. 2026 Sep 15;29(10):117504. doi: 10.1016/j.isci.2026.117504 (PMC13594708; doi:10.1016/j.isci.2026.117504)
Supplement: Supplementary file 1 [file mmc1.pdf]

iScience, Volume *29*

Supplemental information

Transcriptomic characterization of transitional B cells reveals four subsets with perturbed activation profiles in scleroderma

Claire Beesley, Nina Goldman, Gisela Gabernet, Edel Aron, Cankut Çubuk, Myles J. Lewis, Louisa K. James, Gur Yaari, Steven H. Kleinstein, David J. Abraham, Christopher P. Denton, Rizgar A. Mageed, and Voon Ong

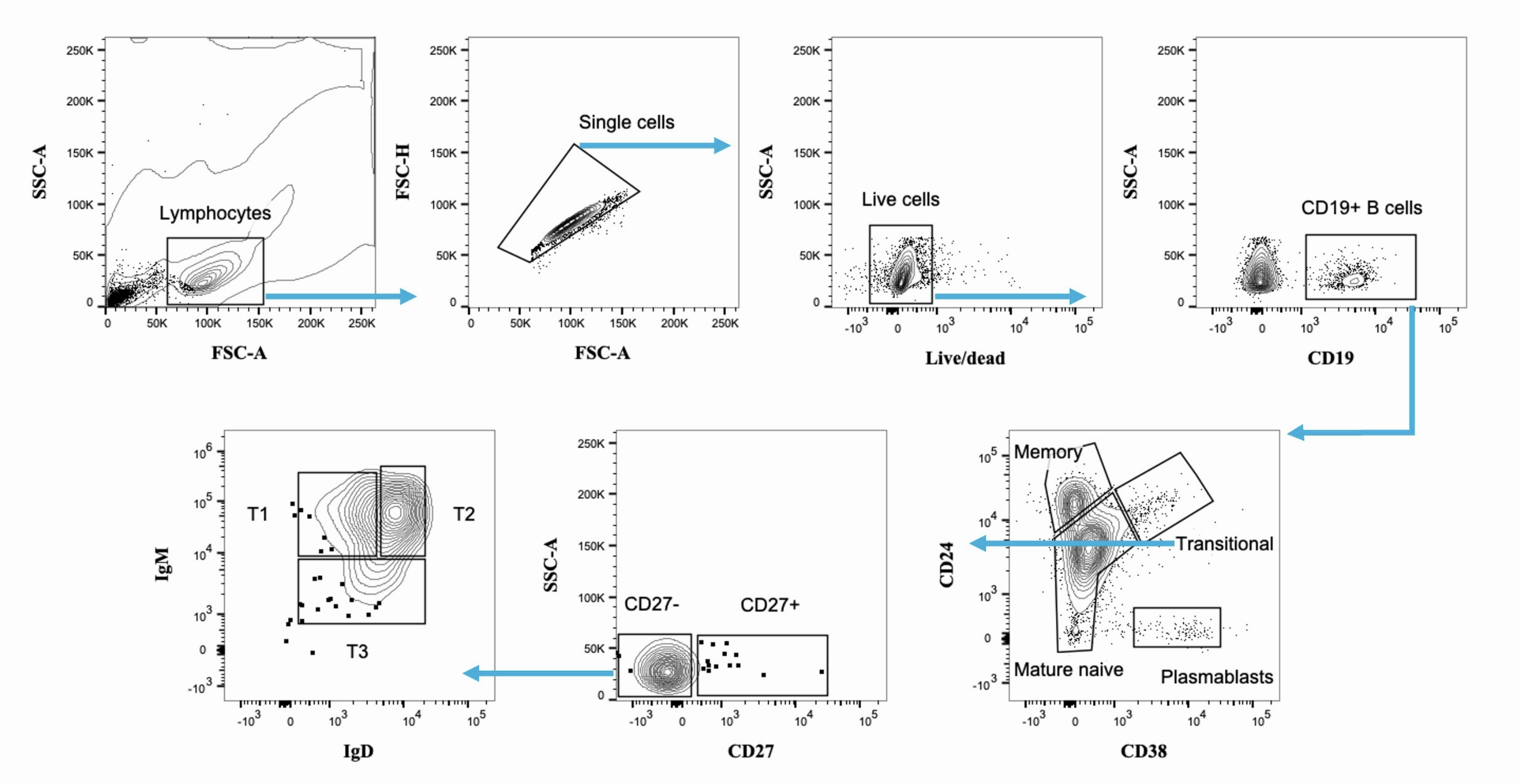

Supplemental Fig 1. Gating strategy used to identify peripheral blood B cell subsets, including the transitional CD27+ subset and CD27- T1, T2 and T3 subsets. Flow cytometry plots shown are from a representative healthy donor.

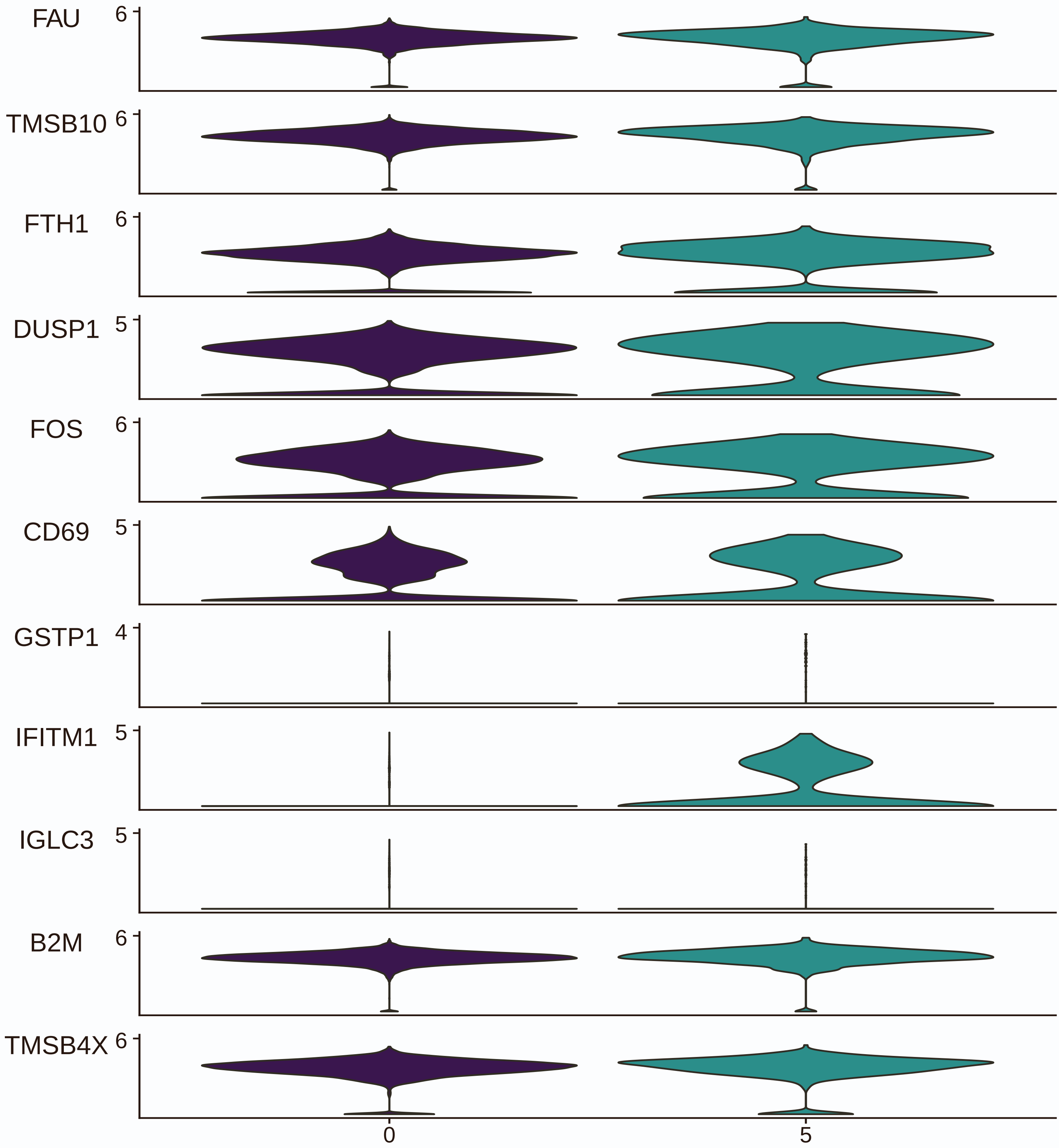

Supplemental Figure 2. Expression of cluster 0 annotation genes in cluster 0 and cluster 5.

A

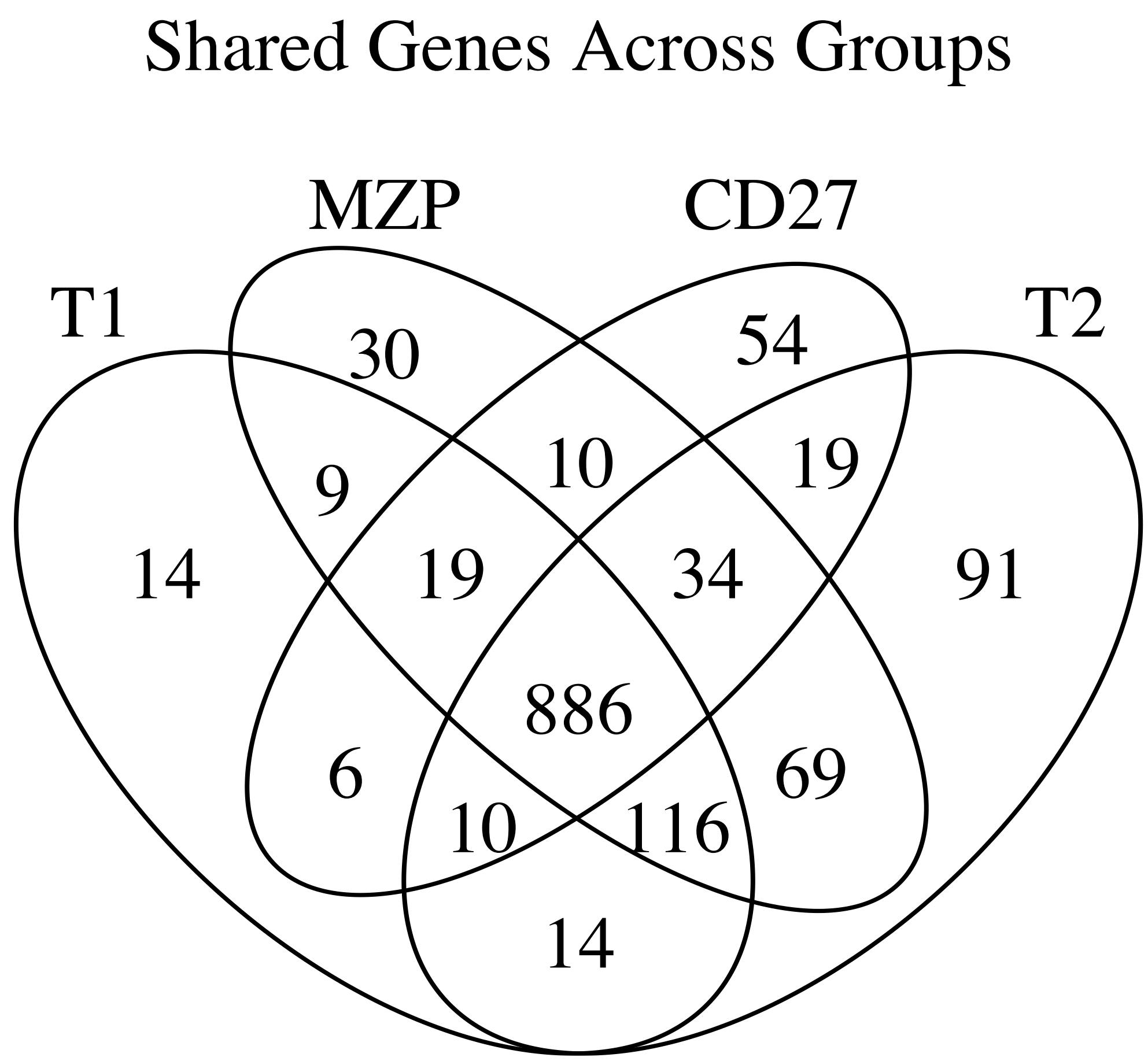

B

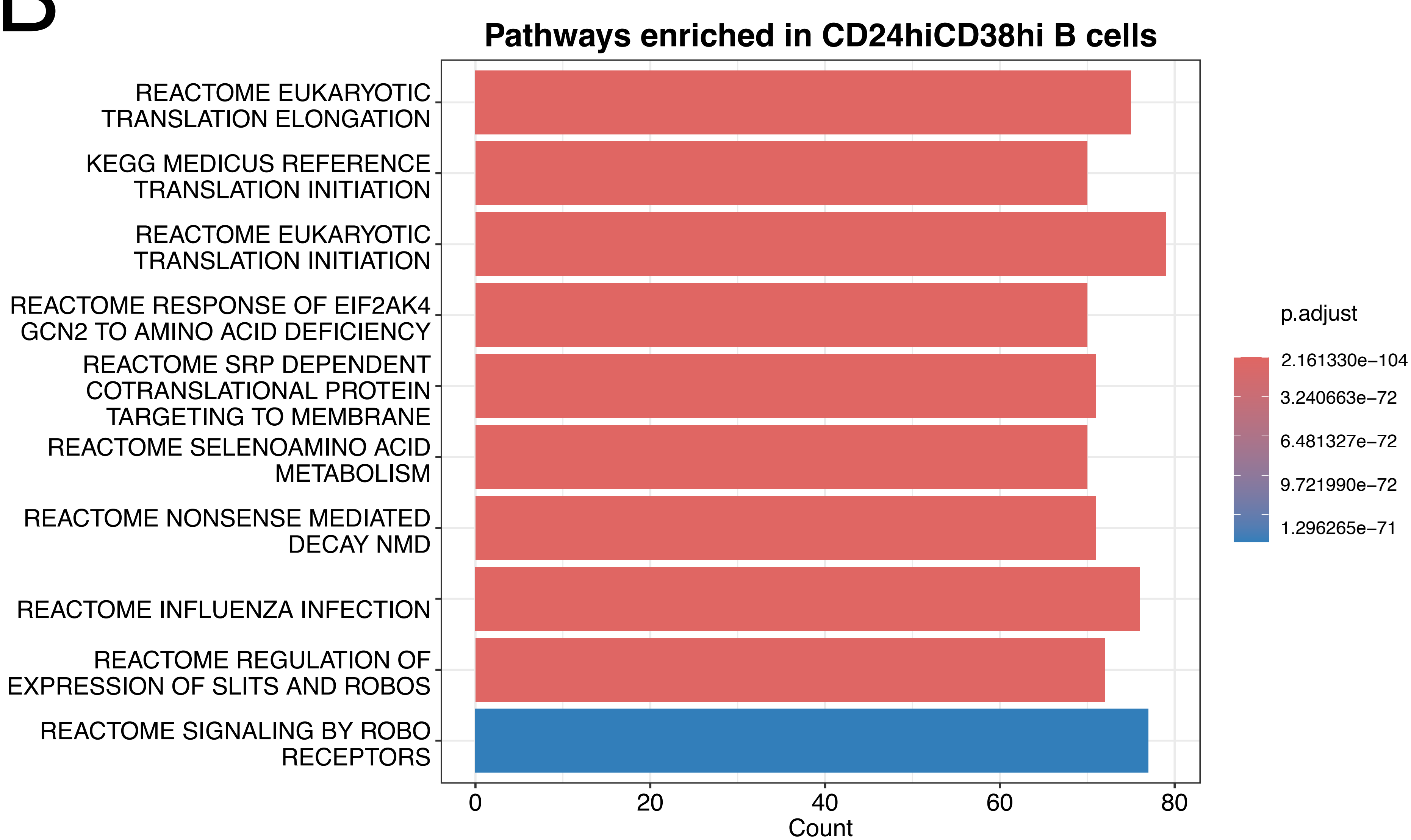

Supplemental Figure 3. Analysis of shared transitional genes in HCs. (A) Venn diagram to show the number of shared genes in T1, MZP, CD27 and T2 B cells. (B) The top 12 pathways identified in the 886 genes shared by all transitional clusters in the Reactome and KEGG database.

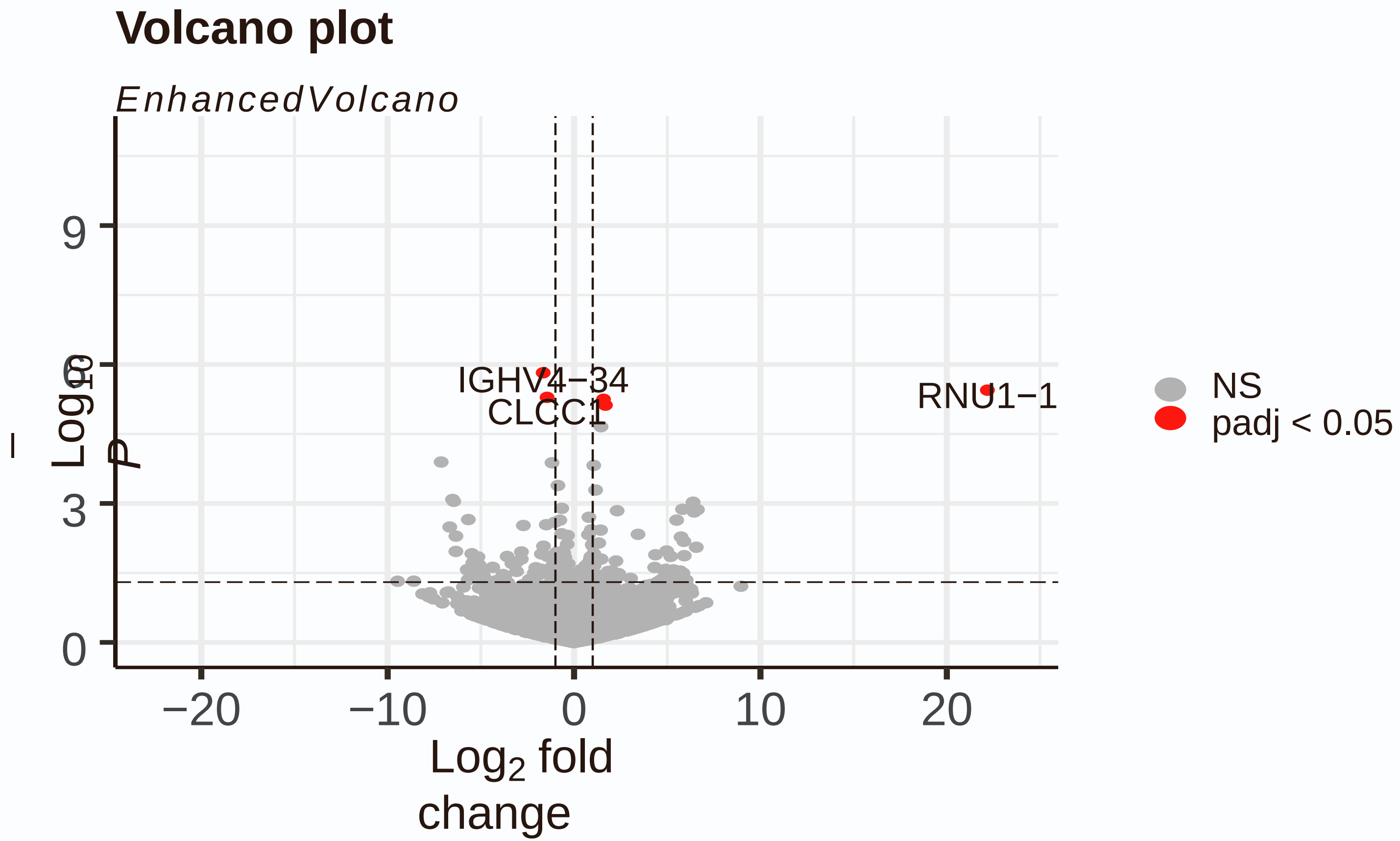

Supplemental Figure 4. Bulk RNA-sequencing analysis of sorted transitional T1 and T2 cells in three SSc patients and two HCs. Differentially expressed genes were identified using DESEQ2.

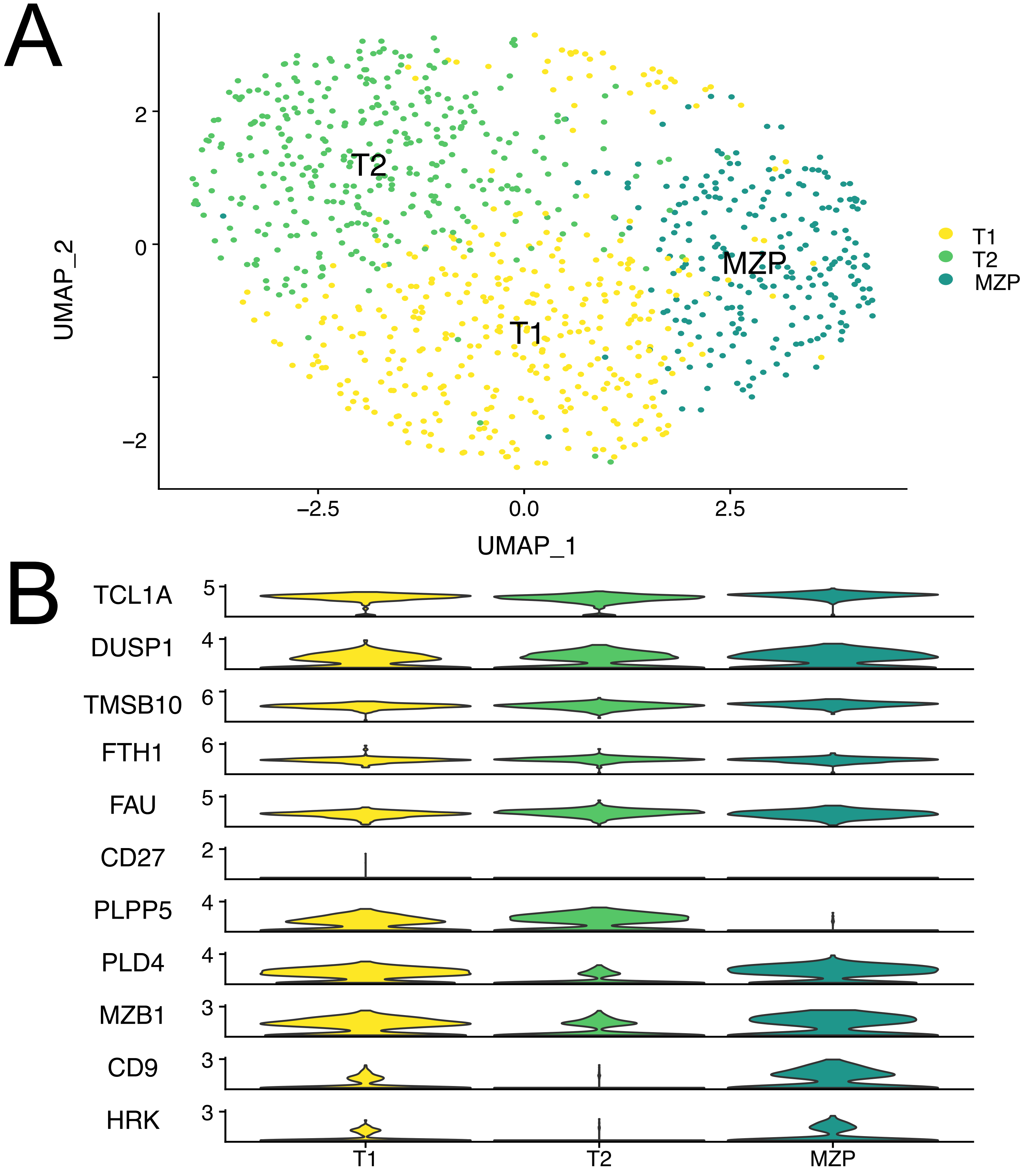

Supplemental Figure 5. Analysis of publicly available transitional B cell transcriptomic data from Stewart et al. (A) UMAP of scRNA-seq data derived from sorted CD19+IgD+CD27-CD10+ transitional B cells isolated from one healthy donor. (B) Expression of key transitional genes in the Stewart et al dataset. Marker genes identified in the literature and through our own analysis of transitional B cells.

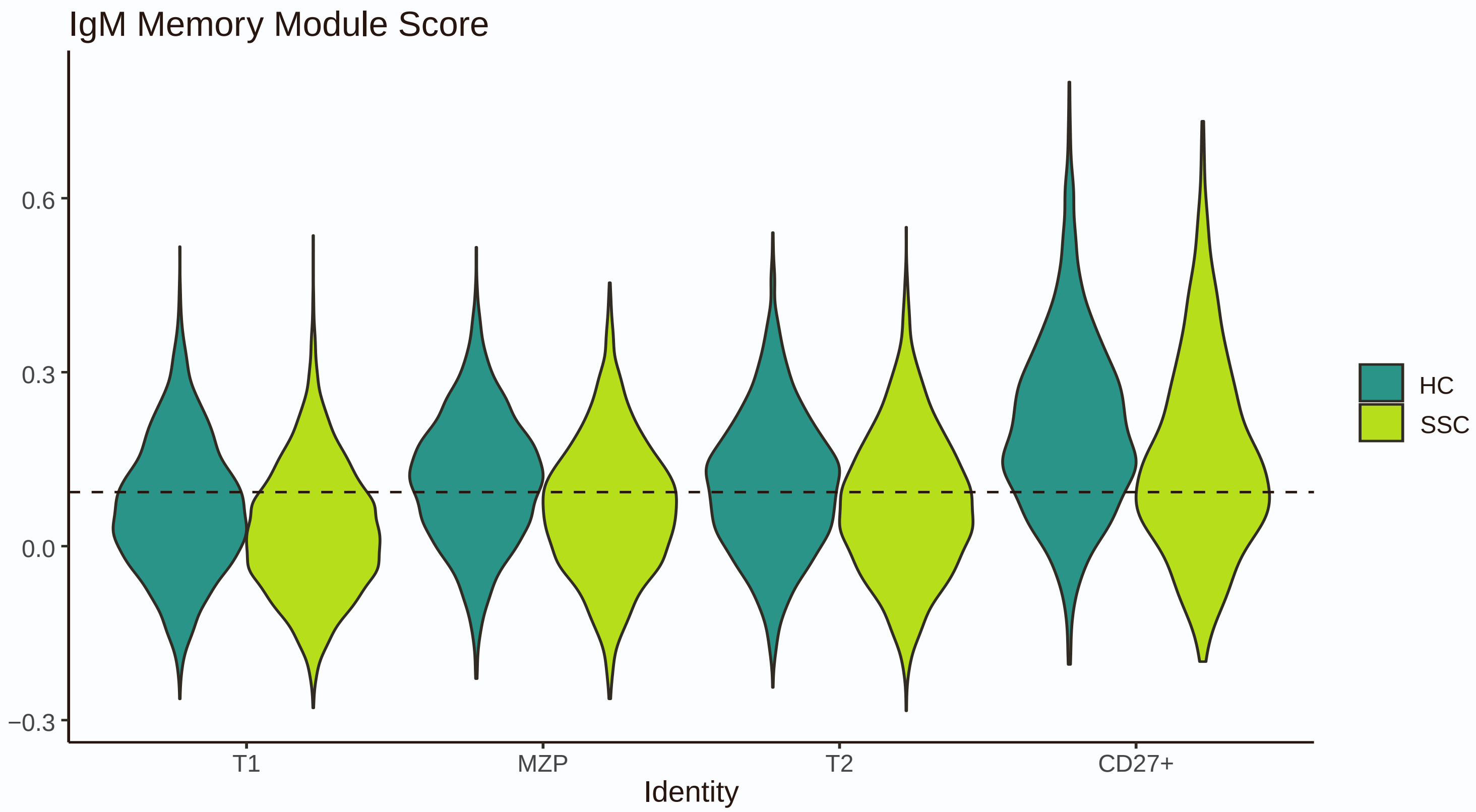

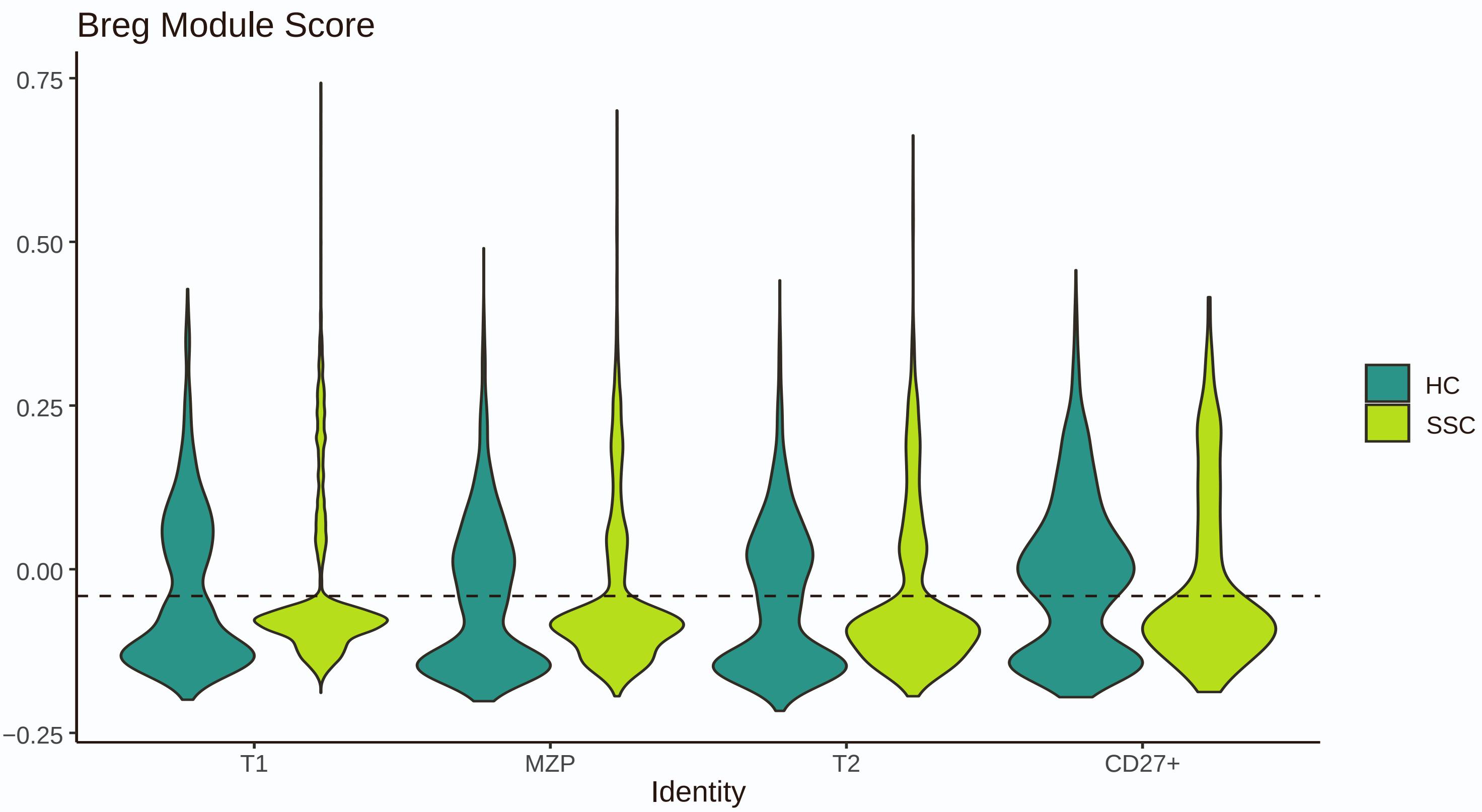

Supplemental Figure 6. Exploratory analysis to interrogate if the CD27+ cluster shares similarity with IgM memory B cells or Bregs. (Top) IgM memory B cell scRNA-seq dataset taken from Stewart et al who sorted for IgM memory B cells (CD19+IgD+CD27+). We analysed this dataset, identifying an IgM memory B cell score of the 62 annotation genes identified (Supplementary Table 2). (Bottom) Breg score taken from Baig et al. For each figure, the line on the graph indicates the mean module score for the HC T1, T2 and MZP clusters.

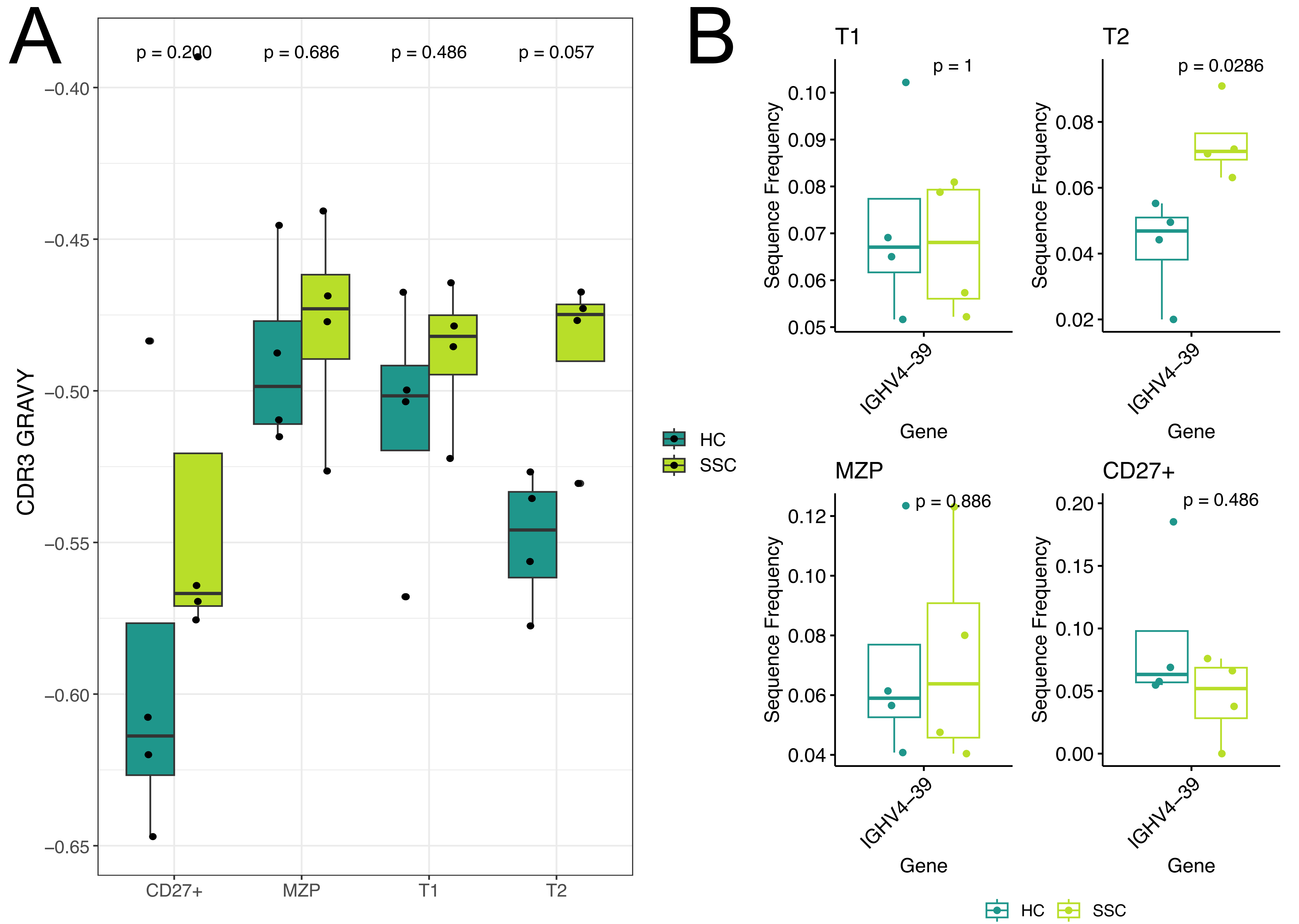

Supplementary Figure 7. Transitional B cell repertoire changes are most evident in the T2 subset in SSc patients compared to healthy donors. (A) CDR3 heavy chain gravy or hydrophobicity amino acid mean values per donor across the clusters. (B) Expression of IGHV4-39 across the transitional clusters. A Wilcoxon rank-sum test was used to compare the means between each group.

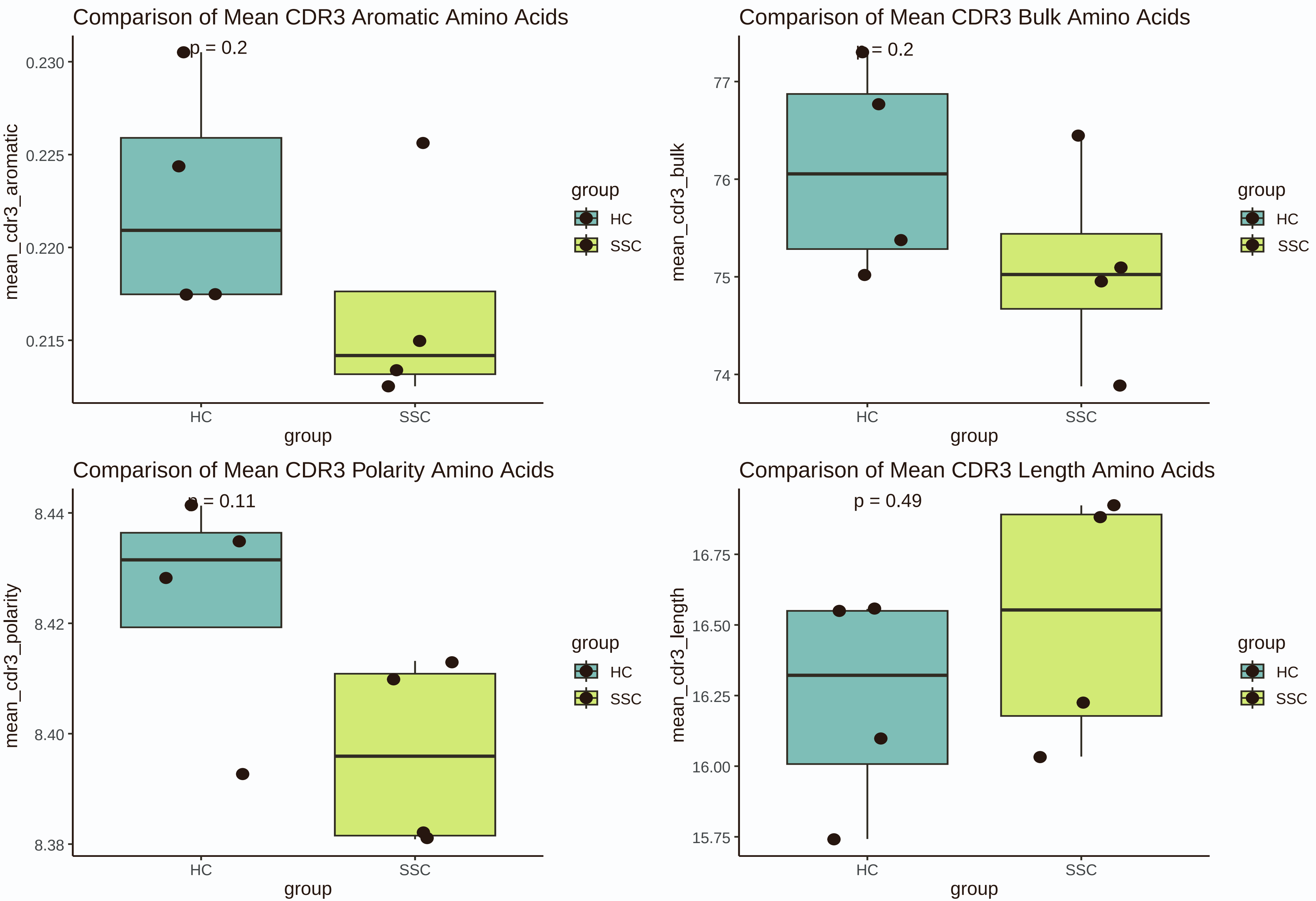

Supplementary Figure 8. Heavy chain CDR3 amino acid properties in transitional B cells from healthy donors and SSc patients. Mean CDR3 properties were calculated per donor. A Wilcoxon rank-sum test was used to compare the means between each group.

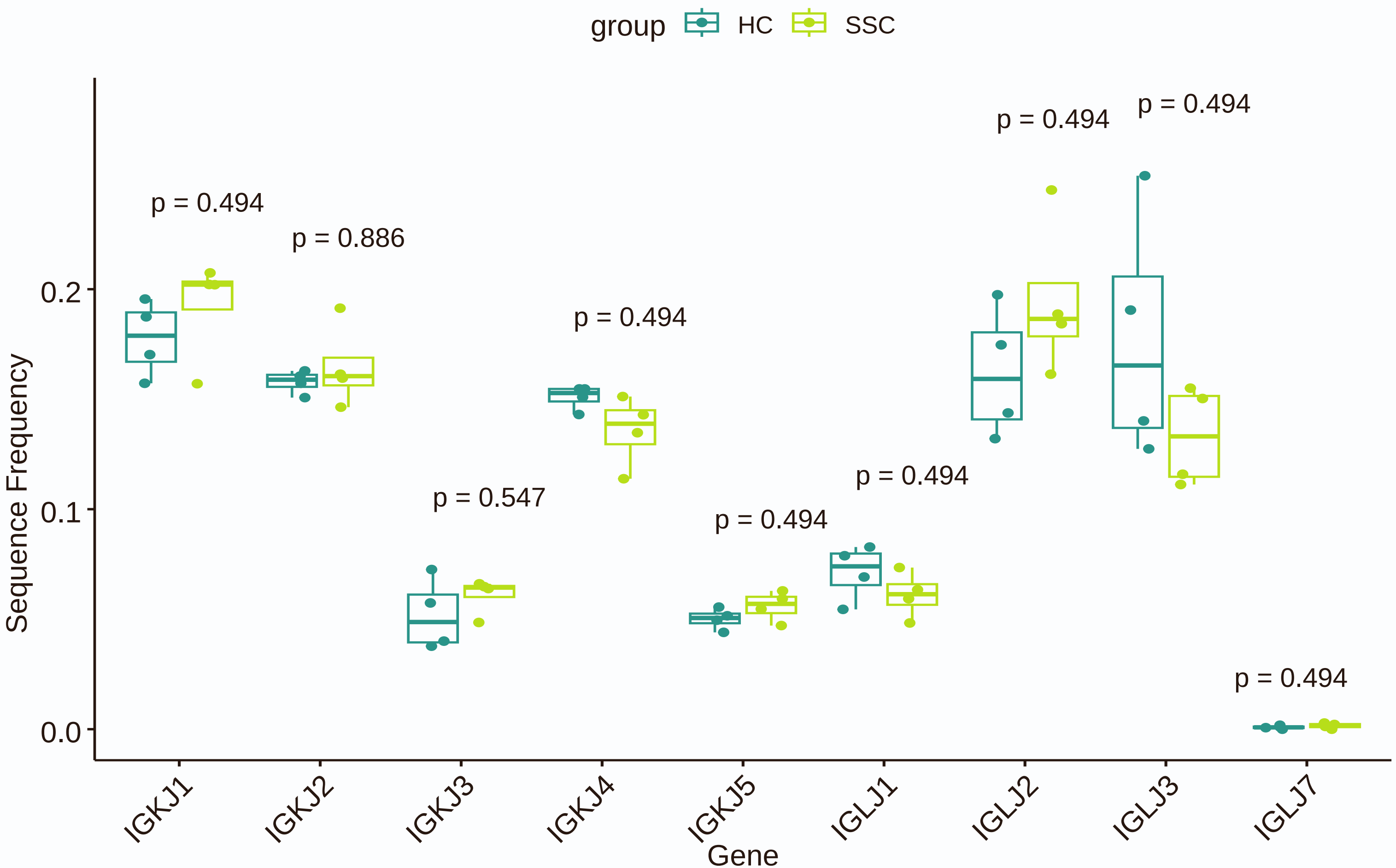

Supplemental Fig 9. Further insights into altered B cell receptor expression in SSc. Boxplots to show the sequence frequency of kappa (IGK) and lambda (IGL) joining genes in HCs and SSc patients. Genes are displayed in their genomic order along the loci. A Wilcoxon rank-sum test was used to compare the means between each group.
